# Modulating Copper(II) Coordination and Antimicrobial Activity: Effects of d-Amino Acid Substitution and *Retro-Inverso* Modification in Human Saliva MUC7 Peptide

**DOI:** 10.1021/acs.inorgchem.5c00438

**Published:** 2025-03-19

**Authors:** Joanna Wątły, Klaudia Szarszoń, Monika Sabieraj, Arian Kola, Robert Wieczorek, Tomasz Janek, Daniela Valensin

**Affiliations:** a Faculty of Chemistry, University of Wrocław, F. Joliot-Curie 14, 50-383 Wrocław, Poland; b Department of Biotechnology, Chemistry and Pharmacy, University of Siena, Via A. Moro 2, 53100 Siena, Italy; c Department Life Science, University of Siena, Via A. Moro 2, 53100 Siena, Italy; d Department of Biotechnology and Food Microbiology, Wrocław University of Environmental and Life Sciences, Chełmońskiego 37, 51-630 Wrocław, Poland; e CIRMMP, Via Luigi Sacconi 6, 50019 Firenze, Italy

## Abstract

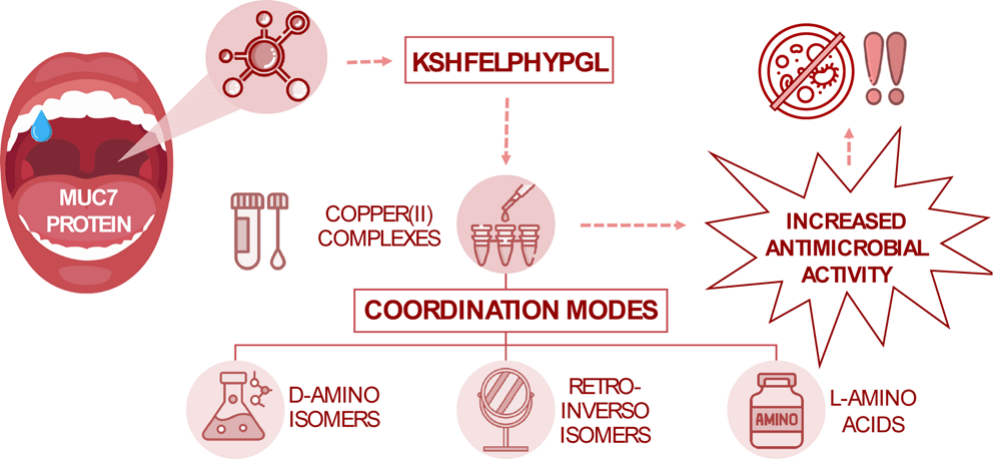

Fragments of MUC7, a salivary protein involved in nonimmune defense, arise from proteolytic cleavage in saliva and exhibit antimicrobial properties. However, their therapeutic use is limited by low stability due to further degradation. To address this, a native MUC7 fragment was modified using d-amino acids and the *retro-inverso* strategy. Given the role of metal ions in enhancing antimicrobial peptides, we analyzed the bioinorganic chemistry of these systems with Cu(II) and assessed their antimicrobial activity against fungal and bacterial strains. This study is the first to explore the correlation between metal binding mode, structure, stability, and antimicrobial activity of *retro-inverso* peptides as well as Cu(II) coordination in such systems. A combination of experimental techniques (potentiometry, mass spectrometry, UV–vis, circular dichroism, electron paramagnetic resonance, and nuclear magnetic resonance spectroscopy) and density functional theory calculations characterized their coordination chemistry. Our results demonstrate that the “standard” enantiomeric exchange and *retro-inverso* modifications of the MUC7 fragment have a minimal effect on the secondary structure and biological activity of the studied peptides and their Cu(II) complexes. However, these modifications significantly influence on the thermodynamic stability of studied systems.

## Abstract 2

Fragments of MUC7, a salivary protein of the nonimmune defense system, exhibit antimicrobial activity but are prone to proteolytic degradation. To enhance stability, the native fragment was modified using d-amino acids and a *retro-inverso* strategy. The coordination chemistry of these peptides with Cu(II) ions was analyzed through experimental and computational methods, revealing a correlation between metal binding, structure, and antimicrobial activity. This study offers novel insights into Cu(II)-*retro-inverso* peptide complexes, highlighting their coordination properties and therapeutic potential.

## Introduction

The increasing prevalence of multidrug-resistant bacteria has intensified interest in antimicrobial peptides (AMPs) as promising therapeutic agents. AMPs offer several advantages, including slower rates of resistance development and unique mechanisms of action. Moreover, their inherent structural versatility allows for various chemical modifications, enabling the design of novel agents with enhanced therapeutic efficacy and safety profiles.^1,2^ The coordination of metal ions can further augment the activity of AMPs as these ions can influence the peptides’ physicochemical properties, such as local charge and structural conformation.^3^

Among the diverse AMPs, those derived from human saliva—including histatins, defensins, cathelicidins, and mucins—are of particular significance for us.^4,5^ These peptides often result from the proteolytic cleavage of precursor proteins present in saliva. For example, histatins, primarily encoded by the HTN1 and HTN3 genes, undergo post-translational modifications or proteolytic digestion to yield various active forms.^6,7^ Another notable example is mucin 7 (encoded by MUC7), from which proteolytic fragments, such as the 51-mer (EGRERDHELRHRRHHHQSPKSHFELPHYPGLLAHQKPFIRKSYKCLHKRCR), 20-mer (LAHQKPFIRKSYKCLHKRCR), and 12-mer (RKSYKCLHKRCR), have been identified as active antimicrobial agents.^8−11^

Despite the potent bioactivity of natural peptides, they are often limited by rapid in vivo clearance, susceptibility to proteolysis, and potential immunogenicity, which may lead to adverse effects.^12^ To overcome these challenges, antimicrobial peptidomimetics have emerged as promising alternative. These synthetic analogues are designed to mimic the cationic charge, hydrophobicity, and amphiphilicity of natural AMPs while offering improved stability and resistance to proteolytic degradation.^13,14^ Peptidomimetics may incorporate non-natural building blocks and chemical modifications to enhance their therapeutic potential.^15,16^

One strategy in peptidomimetic design aimed at overcoming proteolytic vulnerability is the substitution of l-amino acids with d-amino acids-mirror-image counterparts of the naturally occurring l-amino acids, which are stereoisomers resistant to proteolysis. Although d-amino acids share similar chemical and physical properties with l-amino acids, they differ in their ability to rotate circularly polarized light in the opposite direction.^17^ Furthermore, incorporating d-amino acids into the peptide sequence can promote the formation of specific secondary structures and improve metal binding affinity and antimicrobial activity.^18,19^ While l-amino acids are the building blocks of natural proteins synthesized by ribosomes, d-amino acids can be found in some post-translational modifications and in bacterial cell walls. Although human proteins are composed exclusively of l-amino acids, d-amino acids can appear in certain tissues, such as the brain, teeth, eye lens, skin, and bones, as a result of aging.^20^

Another approach that aims to increase the proteolytic stability involves the synthesis of *retro-inverso* (RI) peptides, which feature reversed sequences and chirality relative to their native counterparts. This modification preserves the side-chain orientation and often maintains the overall structural integrity of the peptide.^21^ This technique enables the development of peptidomimetics with enhanced properties compared to those of the original peptides. For example, the reversed chirality of RI peptides makes them less susceptible to proteolytic degradation, leading to longer half-lives, which are crucial in drug design. Additionally, RI peptides often exhibit improved stability and biological activity. Some studies show that RI peptides have higher specificity for molecular targets and better in vivo bioavailability compared to their native forms.^21−23^

**Figure 1:**
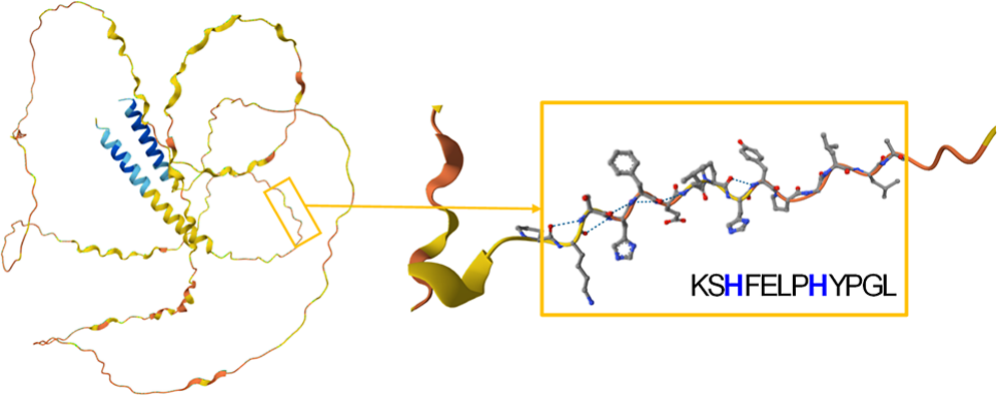
AlphaFold-predicted structure of human MUC7 (UniProtKB: Q8TAX7) with an enlarged fragment of the studied peptide KSHFELPHYGL (native sequence).

In the present study, we employed both modifications: (i) replacement of l-amino acids with d-amino acids and (ii) the *retro-inverso* strategy to design a peptidomimetic analogue of the 12-amino acid fragment of mucin 7 (residues 20–32 of the MUC7 sequence) (Figure 1). This fragment was selected due to its predicted presence in saliva, which directly precedes the well-characterized 20-mer region known for its biological activity.^8^ Additionally, its cationic nature (+4 net charge) and specific binding motifs make it a strong candidate for investigating Cu(II)-mediated antimicrobial activity and for designing proteolytically resistant peptidomimetics.

We aimed to investigate: (i) the differences in Cu(II) coordination between the native and modified peptides; (ii) the impact of metal ion coordination on the secondary structure and antimicrobial activity; and (iii) the influence of peptidomimetic modifications on the secondary structure and antimicrobial efficacy of the studied peptide.

The investigation of metal ion coordination in AMPs is fundamental for understanding their mechanism of action. Metal ions frequently play a critical role in AMP activity through two primary mechanisms: (i) by participating in “nutritional immunity”, where peptides sequester metal ions crucial for pathogen survival and virulence;^24,25^ and (ii) by enhancing the antimicrobial properties of the peptides, often through alterations in their structure or net charge.^26−28^

**Figure 2:**
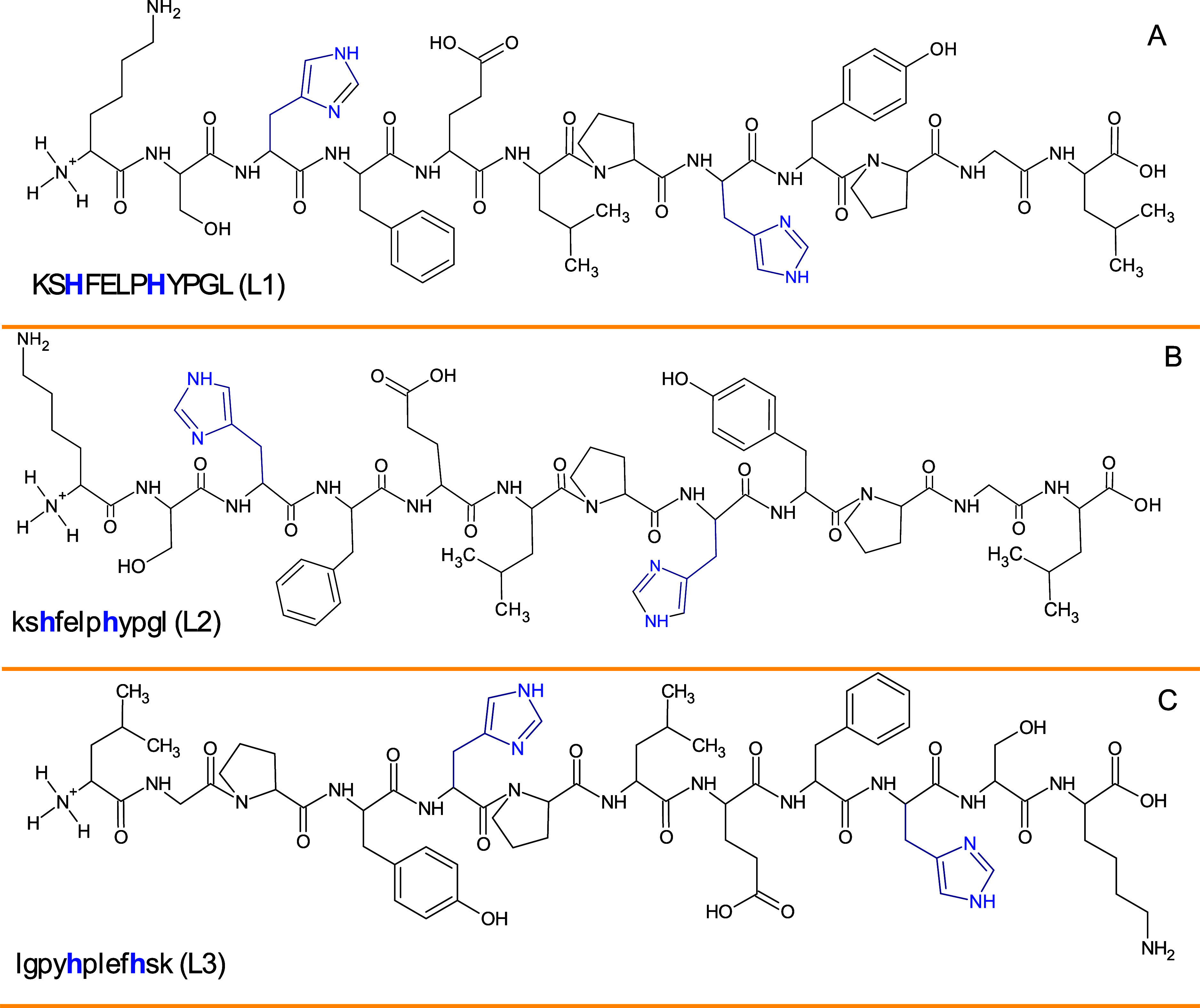
Schematic structure of the 12-amino acid native L-peptide, KSHFELPHYPGL (L1) from the salivary protein MUC7 (A) and its d-amino acid—kshfelphypgl, L2 (B), and *retro-inverso*—lgpyhplefhsk, L3 (C) peptidomimetics. The uppercase letters indicate l-amino acids, and lowercase letters represent d-amino acids.

The studied fragments (Figure 2) consist of 12 amino acid residues, including two histidyl residues, which serve as ideal anchoring sites for the Cu(II) ion. Both the native and d-amino acid-modified sequences feature a characteristic ATCUN motif [amino-terminal Cu(II)- and Ni(II)-binding site], commonly referred to as the “albumin-like binding site”.^29−31^ Notable examples of AMPs containing the ATCUN motif include histatins, shepherin II, and other fragments of mucin 7, such as HHHQSPK.^7,32−35^ In many cases, the coordination of metal ions to the ATCUN motif enhances the antimicrobial activity of peptides.^36,37^ The ATCUN motif comprises a free amino group at the N-terminus, a histidine residue at the third position, and two amide groups between them. This specific arrangement enables the motif to bind Cu(II) and Ni(II) ions with a high affinity. Within a pH range of 4.5–8, the ATCUN motif typically starts to form a 4 N square planar geometry complex with Cu(II), utilizing a donor set consisting of {NH_2_, 2N_am_, N_im_} groups.^29,38,39^

## Experimental Section

### Materials

All the ligands are unprotected peptides: KSHFELPHYPGL (L1, l-amino acid peptide, native peptide), kshfelphypgl (L2, d-amino acid analogue), and lgpyhplefhsk (L3, peptide synthesized by *retro-inverso* strategy, RI) were purchased from KareBay Biochem (USA) (certified purity of 98%, Figure S1A–C) and used without additional purification. The electrospray ionization-mass spectrometry (ESI-MS) samples were prepared by using a mixture of ultrapure methanol (Sigma-Aldrich, St. Louis, Missouri, USA) and water. Cu(ClO_4_)_2_·6H_2_O was an extra-pure product (Sigma-Aldrich, Saint Louis, Missouri, USA). The concentrations of its stock solution was measured using inductively coupled plasma optical emission spectrometry. The carbonate-free stock solution of 0.1 M NaOH (Eurochem BGD, Poland) was standardized potentiometrically with potassium hydrogen phthalate (Sigma-Aldrich, St. Louis, Missouri, USA). The ionic strength (*I*) was adjusted to 0.1 M by addition of NaClO_4_ (Sigma-Aldrich, Saint Louis, Missouri, USA). All samples were prepared by using freshly double-distilled water. For the electron paramagnetic resonance (EPR) measurements, ethylene glycol (Chempur, Poland) was used. High-purity products for biological studies included 4-morpholineethanesulfonic acid (MES) and 4-(2-hydroxyethyl)piperazine-1-ethanesulfonic acid (HEPES), both sourced from Merck Millipore (Darmstadt, Germany). For the nuclear magnetic resonance (NMR) experiments, deuterium oxide (99.90%, Cambridge Isotope Laboratories, Andover, MA, USA), 3-(trimethylsilyl) propionic-2,2,3,3-*d*_4_ acid sodium salt (internal reference standard, Sigma-Aldrich, Saint Louis, Missouri, USA), MES-d13 pH 5.4 (98%, Cambridge Isotope Laboratories, Andover, MA, USA), and phosphate buffer pH 7.4 (Sigma-Aldrich, Saint Louis, Missouri, USA) were used.

### Mass Spectrometric Measurements

High-resolution mass spectra were obtained on a Bruker Compact QTOF (Bruker Daltonik, Bremen, Germany), equipped with an electrospray ionization source with an ion funnel. The instrument was operated in the positive-ion mode. The instrumental parameters were as follows: scan range *m*/*z* 50–2000; dry gas nitrogen; temperature 180 °C; capillary voltage 4500 V; ion energy 5 eV. The Cu(II) complexes [(metal/ligand stoichiometry of 1:1) [ligand]_tot_ = 10^–4^ M] were prepared in a 50:50 MeOH/H_2_O mixture. The samples were infused at a flow rate of 3 μL/min. The instrument was calibrated externally with a Low Concentration Tuning Mix ESI-ToF (Agilent Technologies, Santa Clara, CA, USA). The data were processed using the Bruker Compass DataAnalysis 4.2 program.

### Potentiometry

The pH-metric titrations were conducted in 0.004 M HClO_4_ with an ionic strength of 0.1 M NaClO_4_, utilizing a Metrohm Titrando 809 titrator and a Mettler Toledo InLab Micro combined glass electrode. A thermostabilized glass cell, equipped with a magnetic stirring system, a microburet delivery tube, and an inlet–outlet tube for argon was used for titrations. The solutions were titrated with 0.1 M carbonate-free NaOH. The electrode was calibrated daily for hydrogen ion concentration by titrating HClO_4_ with NaOH in a total volume of 3.0 cm^3^. The standard potential and the slope of the electrode couple were computed by means of the GLEE program.^40^ Stability constants for proton and Cu(II) complexes were determined from titration curves performed over the pH range of 2–11 at a temperature of 25 °C in a total volume of 2.7 cm^3^. The purities and exact concentrations of the ligand solutions were verified using the Gran method.^41^ The ligand concentration was 0.4 mM, with a Cu(II)/ligand ratio of 0.8:1. Stability constant calculations and confirmation of the concentrations determined by the Gran method were carried out using the HYPERQUAD 2006 program.^42^ Standard deviations were calculated using HYPERQUAD 2006 and accounted only for random errors. The constants for the hydrolysis of Cu(II) ions were sourced from the literature.^43,44^ The speciation and competition diagrams were generated using the HYSS program^45^ and visualized with OriginPro 2016.

### UV–Vis, Electron Paramagnetic Resonance, and Circular Dichroism Spectroscopies

The absorption (UV–vis) spectra were recorded on a JASCO V-750 spectrophotometer, and the circular dichroism (CD) spectra were obtained with a JASCO J-1500 CD spectropolarimeter at 25 °C within the 200–800 nm range in a 1 cm quartz cell in the pH range 3.0–12.0. Direct CD measurements (Θ) were converted to mean residue molar ellipticity (Δε) by using the Jasco Spectra Manager. Far-UV CD spectra were recorded in the range of 180–250 nm in a 0.2 mm quartz cell at 25 °C for ligands and complexes at selected pH. The concentrations of solutions utilized for UV–vis and CD spectroscopic analyses were similar to those employed in the potentiometric experiments. The pH of the samples was adjusted by adding appropriate amounts of concentrated NaOH and HClO_4_ solutions as necessary. EPR spectra were acquired at liquid nitrogen temperature by utilizing a Bruker ELEXSYS E500 CW-EPR spectrometer operating at a band frequency of 9.5 GHz. The ligands under investigation were dissolved in aqueous solutions of HClO_4_ acid at *I* = 0.1 M (NaClO_4_), with the addition of ethylene glycol (30%) as a cryoprotectant. The concentration of copper ions was 0.001 M, and the metal/ligand ratio was 0.8:1. Measurements were conducted over the pH range 3.0–11.0. The obtained EPR spectra were analyzed to determine the EPR parameters, which characterize the molecular and electron structures of the copper complexes, employing a simulation method. This involved identifying the best fit between the theoretical and the experimental spectra. The theoretical (simulated) EPR spectra were generated using WinEPR SimFonia software, version 1.2 (Bruker), using the appropriately selected spin Hamiltonian EPR parameters for *S* = 1/2, including the diagonal components of the tensors: *g* (*g*_∥_ = *g*_*z*_, *g*_⊥_ = *g*_*x*_ = *g*_*y*_) and A—interaction of an unpaired electron of copper(II) with a nuclear spin of copper, *I*(^63,65^Cu) = 3/2, (*A*_∥_ = *A*_*z*_, *A*_⊥_ = *A*_*x*_ = *A*_*y*_). The characterization of the various species generated in the solution involved comparing the observed wavelength of maximum absorption in the visible spectra at a specific pH value with the λ_max_ value reported in the literature.^46−53^ OrginPro 2016 was used to process and visualize the obtained spectra.

### Density Functional Theory (DFT) Calculations

Computational methods of theoretical chemistry have been used as valuable tools to predict the structure and stability of ligands and complexes.^54,55^ Molecular orbital studies of 1:1 complexes of Cu(II) cations with the KSHFELPHYPGL (L1) and kshfelphypgl (L2) ligands were performed at the DFT level of theory. The initial structure of the peptide for DFT calculations was generated based on the amino acid sequence after a 75 ps simulation at 300 K, without cutoffs, using the BIO + implementation of the CHARMM force field. DFT calculations were carried out with Gaussian 16C.01^56^ software suite, employing the ωB97X-D long-range corrected hybrid density functional with damped atom–atom dispersion corrections,^57^ and a double-ζ 6–31G (d,p) basis set with polarization functions. All structures presented were fully optimized, and all complexes studied were found to be thermodynamically stable.

### Nuclear Magnetic Resonance Spectroscopy

NMR spectra were recorded at 14.1 T using a Bruker Avance III 600 MHz spectrometer and using a 5 mm BBI (Broad Band Inverse) probe. The temperature was set and maintained to 298 K with an accuracy of ± 0.1 K. The residual water signal was suppressed through excitation sculpting, employing a 2 ms selective square pulse on water. All samples were prepared in a mixture of 90% H_2_O and 10% D_2_O (99.90% purity from Cambridge Isotope Laboratories) with addition of deuterated MES-*d*_13_ 20 mM buffer, pH 5.4 (Cambridge Isotope Laboratories) or phosphate 20 mM buffer, pH 7.4 (Sigma-Aldrich). 3-(Trimethylsilyl)propionic-2,2,3,3-*d*_4_ acid sodium salt (Sigma-Aldrich) was used as an internal reference standard. Proton resonance assignment was achieved using 2D ^1^H–^1^H total correlation spectroscopy (TOCSY) and nuclear overhauser effect spectroscopy (NOESY) experiments conducted with standard pulse sequences. Data processing and analysis were completed using Bruker TOPSPIN 3.6.5 program. The samples of the analyzed complexes were prepared by adding Cu(II) (at different M(II)/L molar ratios) to solutions of a 0.5 mM ligand in appropriate buffer, followed by adjusting the pH to 5.4 and 7.4, as needed. The NMR spectra recorded both in the presence and absence of metal ion identified specific metal-binding sites, and the combination of all methods used provided insights into the coordination geometries.

### *In Vitro* Antimicrobial Activity of Peptides and Peptide-Metal Ion Systems

The antimicrobial properties of the three peptides and their Cu(II) complexes were evaluated against several pathogenic strains that affect humans. This included four reference strains obtained from the American Type Culture Collection (ATCC), specifically *Escherichia coli* 25922, *Pseudomonas aeruginosa* 15442, *Enterococcus faecalis* 29212, and *Staphylococcus aureus* 25923. Additionally, two strains from the Polish Collection of Microorganisms (PCM) were tested: *Streptococcus mutans* 2502 and *Streptococcus sanguinis* 2335, along with *Candida albicans* SC5314, used for antimicrobial activity testing.^58^*E. coli* ATCC 25922, *P. aeruginosa* ATCC 15422, *E. faecalis* ATCC 29212, and *S. aureus* ATCC 25923 were grown at 37 °C in Mueller–Hinton broth (MHB) supplied by Merck Millipore, Darmstadt, Germany. *S. mutans* PCM 2502 and *S. sanguinis* PCM 2335 were cultured in Brain Heart Infusion (BHI) broth, also from Merck Millipore (Darmstadt, Germany), and incubated anaerobically (85% N_2_, 10% H_2_, and 5% CO_2_) overnight at 37 °C. *C. albicans* SC5314 was grown aerobically at 37 °C in yeast peptone dextrose (YPD) broth from A&A Biotechnology (Gdańsk, Poland).

### Bacterial Susceptibility Assay

The minimal inhibitory concentrations (MICs) of the peptides/complexes were assessed by employing the serial broth microdilution technique.^59^ In brief, two-fold serial dilutions of each peptide/Cu(II) complex (1:1 molar ratio) were made in MHB, BHI, and YPD broth, each buffered with either 10 mM MES (pH 5.4) or 10 mM HEPES (pH 7.4) (both from Merck Millipore, Darmstadt, Germany) in volumes of 100 μL in 96-well flat-bottom microtiter plates (Sarstedt, Nümbrecht, Germany). The final concentrations of the peptides/complexes ranged from 7.8 to 500 μg/mL. Each well was inoculated with 1 μL of a 24 h microorganism culture, resulting in a final cell density of 5 × 10^7^ cfu/mL. Negative control and growth control wells did not include the tested compounds. Copper ion (10 μg/mL) served as negative controls as they exhibited no antimicrobial effects. Additional controls included bacteria and *C. albicans* incubated with metal ions. The microplates were incubated for 24 h at 37 °C for *E. coli* ATCC 25922, *P. aeruginosa* ATCC 15422, *E. faecalis* ATCC 29212, *S. aureus* ATCC 25923, and *C. albicans* SC5314. Two oral bacteria strains, *S. mutans* PCM 2502 and *S. sanguinis* PCM 2335, were incubated anaerobically at 37 °C (85% N_2_, 10% H_2_, and 5% CO_2_), and OD_600_ was measured after 72 h using a microplate reader (Spark, Tecan Trading AG., Switzerland). The MIC end point was determined as the lowest concentration that led to complete (100%) inhibition of growth. All assays were conducted in triplicate.

## Results and Discussion

### Deprotonation Constants

Based on a series of potentiometric titrations, seven deprotonation constants (p*K*_a_) were determined for L1 and L2 peptides and six for L3 peptide (Table 1). For the L2 peptide, the deprotonation constant for the C-terminus was not determined since this group most likely deprotonated at a lower pH (below the standard operating range of the electrode). Distribution diagrams for the species of studied ligands are presented in Figure S2. Additionally, a clear difference in the p*K*_a_ value of the N-terminus (above one unit) was observed for this peptide (L2) in comparison to the native (L1) and the *retro-inverso* (L3) peptides (the differences in the shape of the potentiometric titration curves are also visible at alkaline pH values for the studied L1 and L2 peptides, Figure S3). The abovementioned differences observed for L2 peptide are most likely caused by the spatial arrangement of terminal amino-acid residues (Figure 2), which may affect the p*K*_a_ values. The other deprotonation constants recorded for all three ligands are very close to each other (Table 1) and are also consistent with those reported in the literature for similar systems.^32,49,50,60−62^ The amide protons of the peptide backbone remain undissociated within the pH range investigated through potentiometry as they are too weakly acidic to be released spontaneously. However, they can be displaced by Cu(II) ions at the appropriate pH levels.^46^

**Table 1:** Deprotonation Constants (p*K*_a_) for L1, L2, and L3 Peptides and Stability Constants (log β) for Their Complexes with Cu(II) Ions in Aqueous Solution of 4 mM HClO_4_ with *I* = 0.1 M NaClO_4_ at 25 °C. *C*_L_ = 0.4 mM; Molar Ratio M/L–0.8:1^a^

KSHFELPHYPGL L1				kshfelphypgl L2				lgpyhplefhsk L3			
species	log βjk^b^	p*K*_a_^c^	residue	species	log βjk^b^	p*K*_a_^c^	residue	species	log βjk^b^	p*K*_a_^c^	residue
[H_7_L]^4+^	47.47(4)	2.91	COOH	[H_7_L]^4+^			COOH	[H_7_L]^4+^	48.22(3)	3.01	COOH
[H_6_L]^3+^	44.56(2)	3.82	Glu	[H_6_L]^3+^	46.70(3)	3.99	Glu	[H_6_L]^3+^	45.21(2)	3.98	Glu
[H_5_L]^2+^	40.74(2)	5.98	His	[H_5_L]^2+^	42.71(3)	6.21	His	[H_5_L]^2+^	41.23(1)	6.18	His
[H_4_L]^+^	34.76(1)	6.72	His	[H_4_L]^+^	36.50(3)	6.89	His	[H_4_L]^+^	35.05(1)	6.83	His
[H_3_L]	28.04(2)	7.55	H_3_N ^+^	[H_3_L]	29.61(2)	8.78	H_3_N ^+^	[H_3_L]	28.22(1)	7.81	H_3_N ^+^
[H_2_L]^−^	20.49(1)	9.78	Tyr	[H_2_L]^−^	20.83(1)	10.07	Tyr	[H_2_L]^−^	20.41(1)	9.72	Tyr
[HL]^2–^	10.71(1)	10.71	Lys	[HL]^2–^	10.76(2)	10.76	Lys	[HL]^2–^	10.69(1)	10.69	Lys

species	log β_jk_^d^	p*K*_a_^e^		species	log β_jk_^d^	p*K*_a_^e^	species	log β_jk_^d^	p*K*_a_^e^	
							[CuH_3_L]^2+^	35.30(3)		
[CuH_2_L]^+^	31.57(1)			[CuH_2_L]^+^	33.52(1)		[CuH_2_L]^+^	30.57(1)	4.73	
[CuHL]	26.97(1)	4.60		[CuHL]	28.34(1)	5.18	[CuHL]	23.28(3)	6.75	
[CuL]^−^	20.13(2)	6.84		[CuL]^−^	18.97(2)	9.37	[CuL]^−^	14.40(5)	9.42	
[CuH_–1_L]^2–^	10.28(3)	9.85		[CuH_–1_L]^2–^	8.76(2)	10.21	[CuH_–1_L]^2–^	4.41(4)	10.09	
[CuH_–2_L]^3–^	–0.47(3)	10.75		[CuH_–2_L]^3–^	–2.23(2)	10.99	[CuH_–2_L]^3–^	–6.70(6)	11.01	
							[CuH_–3_L]^4–^	–18.0(5)	11.30	

a The standard deviations are reported in parentheses as uncertainties on the last significant figure.

b Constants are presented as cumulative log β_jk_ values. β(H_j_L_k_) = [H_j_L_k_]/([H]^j^[L]^k^), in which [L] is the concentration of the fully deprotonated peptide.

c p*K*_a_ values of the peptides were derived from cumulative constants: p*K*_a_ = log β(H_j_L_k_)–log β(H_j–1_L_k_).

d Cu(II) stability constants are presented as cumulative log β_ijk_ values. L stands for a fully deprotonated peptide ligand that binds Cu(II) ion: β(M_i_H_j_L_k_) = [M_i_H_j_L_k_]/([M]^i^[H]^j^[L]^k^), where [L] is the concentration of the fully deprotonated peptide.

e p*K*_a_ = log β (M_i_H_j_+1*L*_k_)–log β(M_i_H_j_*L*_k_).

**Table 2:** Metal–Ligand Distances in Angstroms

	Cu(II)–KSHFELPHYPGL (L1)	Cu(II)–kshfelphypgl (L2)
N···Cu(II) (K1)	2.862	2.925
N···Cu(II) (S2)	1.891	2.311
N···Cu(II) (H3)	1.976	1.877
N···Cu(II) (H3 imidazole)	1.881	1.864

### Cu(II) Complexes

The characterization of Cu(II) complexes has been performed using ESI-MS, potentiometry, UV–vis and EPR, CD (including far-UV region for secondary structure determination), and NMR spectroscopies.

All the used techniques indicate that the three studied peptides form mononuclear complexes with the Cu(II) ions under given conditions, neither polynuclear complexes nor bis-complexes were detected by mass spectrometry or potentiometry (see below).

The most intense signals (*m*/*z*) of each systems were identified and assigned to the corresponding species (Table S1). The signals and isotopic distributions in the experimental and simulated spectra for the selected signals are consistent, confirming the accuracy of the interpretation (Figures S4–S6). The additional signals observed in the spectra primarily correspond to the sodium and potassium adducts of studied ligands and complex species as well as impurities remaining in the measuring instrument.

### Cu(II)–L1 (KSHFELPHYPGL) System

**Figure 3:**
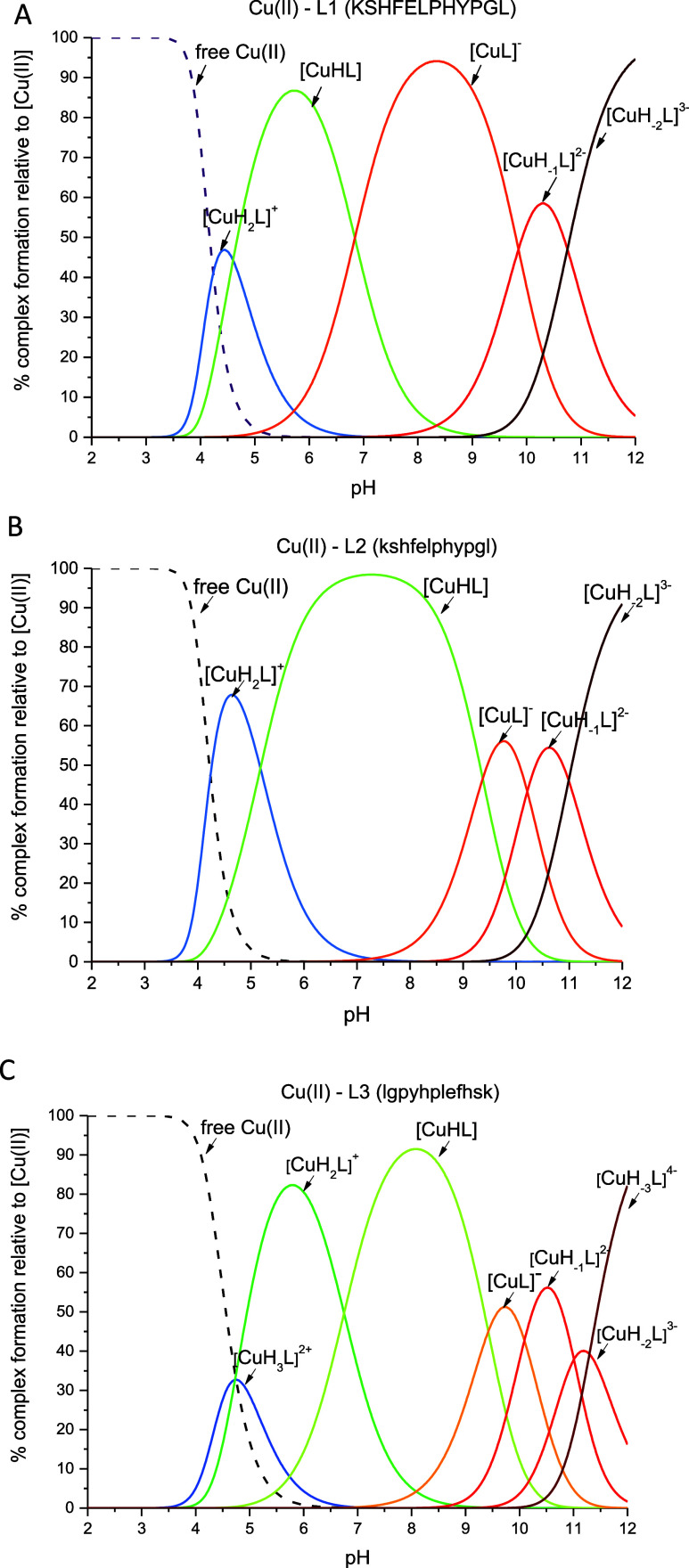
Representative distribution diagram for (A) Cu(II)–L1 (KSHFELPHYPGL); (B) Cu(II)–L2 (kshfelphypgl) and (C) Cu(II)–L3 (lgpyhplefhsk) systems in aqueous solution of 4 mM HClO_4_ with *I* = 0.1 M NaClO_4_ dependent on pH values. *C*_L_ = 0.4 mM; molar ratio M/L–0.8:1. *T* = 25 °C.

**Figure 4:**
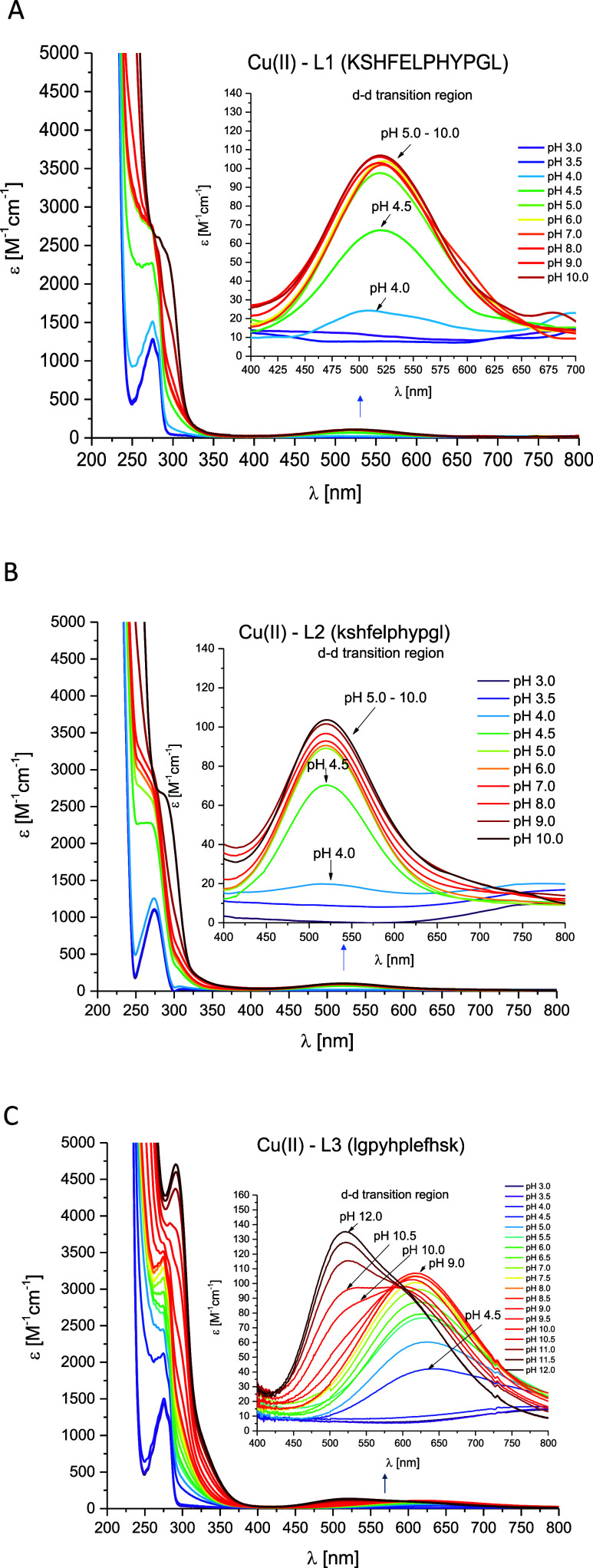
pH-dependent UV–vis absorption spectra for (A) Cu(II)–L1 (KSHFELPHYPGL); (B) Cu(II)–L2 (kshfelphypgl) and (C) Cu(II)–L3 (lgpyhplefhsk) systems in aqueous solution of 4 mM HClO_4_ with *I* = 0.1 M NaClO_4_. Optical path length of 1 cm. *C*_L_ = 0.4 mM; molar ratio M/L–0.8:1. *T* = 25 °C.

**Figure 5:**
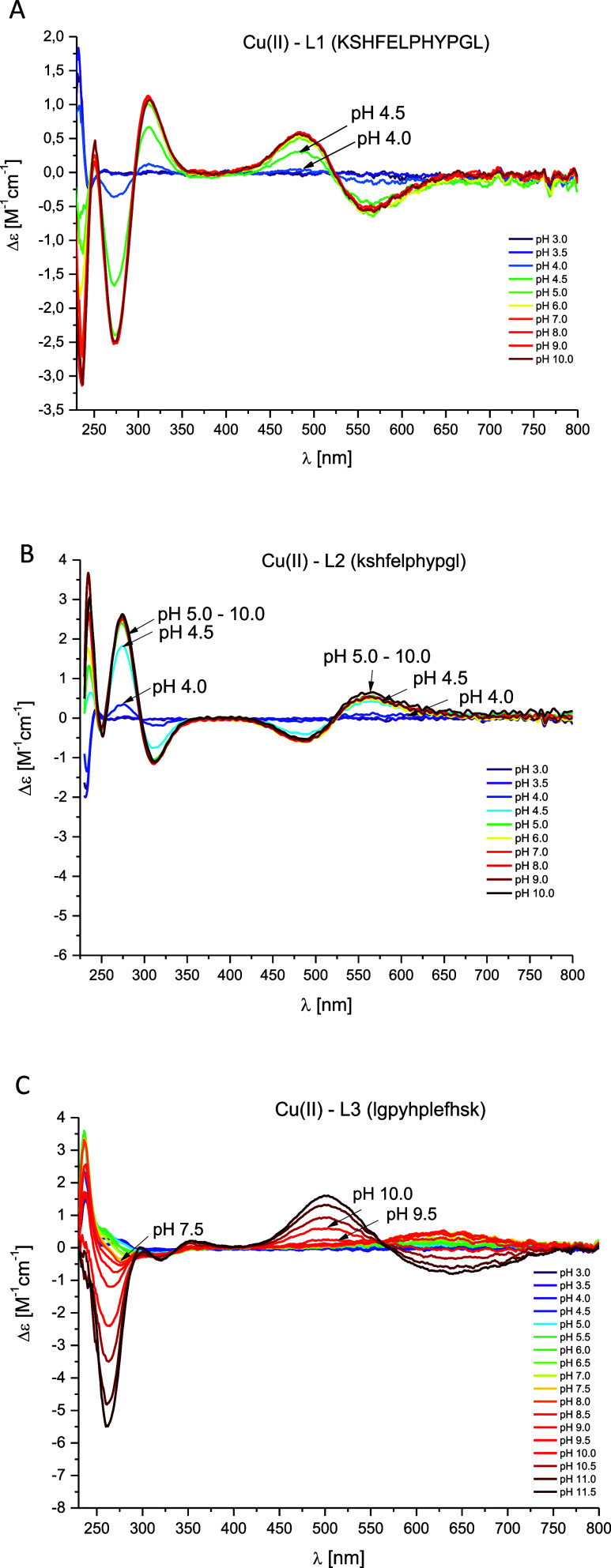
pH-dependent CD spectra for (A) Cu(II)–L1 (KSHFELPHYPGL); (B) Cu(II)–L2 (kshfelphypgl) and (C) Cu(II)–L3 (lgpyhplefhsk) systems in aqueous solution of 4 mM HClO_4_ with *I* = 0.1 M NaClO_4_. Optical path length of 1 cm. *C*_L_ = 0.4 mM; molar ratio M/L–0.8:1. *T* = 25 °C.

Potentiometric measurements revealed that Cu(II) starts interacting with the L1 peptide above pH 3.5 (Figure 3A) forming a [CuH_2_L]^+^ complex species where the metal ion is probably bound by the {1N_im_, 1NH_2_, 1N_am_} donor set. The coordination mode is supported by spectroscopic data: d–d band in UV–vis spectra at 550 nm (Figure 4A) and EPR parameters (*A*_∥_ = 189 G, *g*_∥_ = 2.24) (Figure S7A) indicate a 3 N complex. The strong band at 245 nm in the CD spectrum confirms the coordination of the N_im_ to the Cu(II) ion,^63^ while the bands at 265 and 312 nm indicate the involvement of nitrogen atoms from the N-terminus and the amide group,^63^ respectively (Figure 5A). The next complex species, [CuHL], dominates at pH 5.6 and is probably associated with coordination of the next amide nitrogen forming specific, albumin-like coordination mode with {1N_im_, 1NH_2_, 2N_am_} donor set. 4 N coordination is confirmed by d–d band in UV–vis spectra at 520 nm (Figure 4A) and EPR parameters (*A*_∥_ = 205 G, *g*_∥_ = 2.18, Figure S5A). Additionally, the occurrence of two characteristic bands with (i) positive at 475 nm and (ii) negative at 575 nm Cotton effects above pH 4 in the CD spectra (Figure 5A) suggests square planar geometry, typical for Cu(II) complexes with the ATCUN motif.^64^ This binding mode remains unchanged in the next three complex species: [CuL]^−^, [CuH_–1_L]^2–^, and [CuH_–2_L]^3–^, which are probably associated with deprotonation on nonbinding His residue (p*K*_a_ 6.84 in the complex and p*K*_a_ 6.72 in the ligand), Tyr residue (p*K*_a_ 9.85 in the complex and p*K*_a_ 9.78 in the ligand), and Lys residue (p*K*_a_ 10.75 in the complex and p*K*_a_ 10.71 in the ligand), respectively (Table 1).

### Cu(II)–L2 (kshfelphypgl) System

Coordination of Cu(II) ions to L2 peptide proceeds in a very similar way as observed in the case of the Cu(II)–L1 system descried above, which could be expected; both peptides have the same amino acid sequence with the ATCUN motif. UV–vis (Figure 4B) and EPR (Figure S7B) spectroscopies suggest that the 4 N coordination starts to occur from pH around 4 (ε_max_ at λ = 519 nm in the UV–vis spectrum and *A*_∥_ = 190.8 and *g*_∥_ = 2.20 parameters in the EPR spectrum), the same as in the Cu(II)–L1 system. In the CD spectra (Figure 5B), characteristic charge-transfer bands for N_im_ → Cu(II) (at 236 nm), N_am_ → Cu(II) (at 312 nm) and NH_2_ → Cu(II) (at 274 nm)^63^ as well as bands typical for the square planar geometry (at 486 and 562 nm) are also observed, wherein, the cotton effect values assigned to them have the opposite sign in comparison to the spectra for the Cu(II)–L1 system due to the nature of d-amino acids—they rotate circularly polarized light in the opposite direction than l-amino acids.^65,66^

However, the presence of l- and d-amino acids caused some differences to appear, such as the dominance of individual complex species in solution at a given pH value (Figure 3A,B). It was observed that at near-physiological pH in the Cu(II)–L1 system, the dominant complex species is [CuL]^−^, while in the Cu(II)–L2 system, the dominant is [CuHL]. This situation is most likely reflected by the UV–vis spectra for these systems (Figure 4A,B). In the first case, Cu(II)–L1, from pH above 5 no changes in the band intensity at about 520 nm are observed (Figure 4A), while in the case of the second system, this invariance is observed in the pH ranges 5–7, after which at pH 8 (where the [CuL]^−^ complex species starts to appear in the solution), there is a slight increase in the molar extinction coefficient value, which remains unchanged up to strongly alkaline pH. For the [CuL]^−^ complex species, significantly different p*K*_a_ values are assigned in each of the systems: (i) p*K*_a_ = 6.84 in the Cu(II)–L1 system and (ii) p*K*_a_ = 9.37 in the Cu(II)–L2 system. The p*K*_a_ = 9.37 value should be assigned to the deprotonating nonbinding His residue; however, this would suggest an increase of its value by 2.48 units (p*K*_a_ = 6.89 in the ligand), which has not been found in the literature so far. The differences at alkaline pH are also observed in the shape of the titration curves, where the Cu(II)–L1 and Cu(II)–L2 systems are compared (Figure S8). Such behavior has not been previously reported in the literature for Cu(II) complexes with peptides containing the ATCUN motif (composed of both l-amino acids and their d-amino acid counterparts)—typically, no differences have been observed.^67,68^

**Figure 6:**
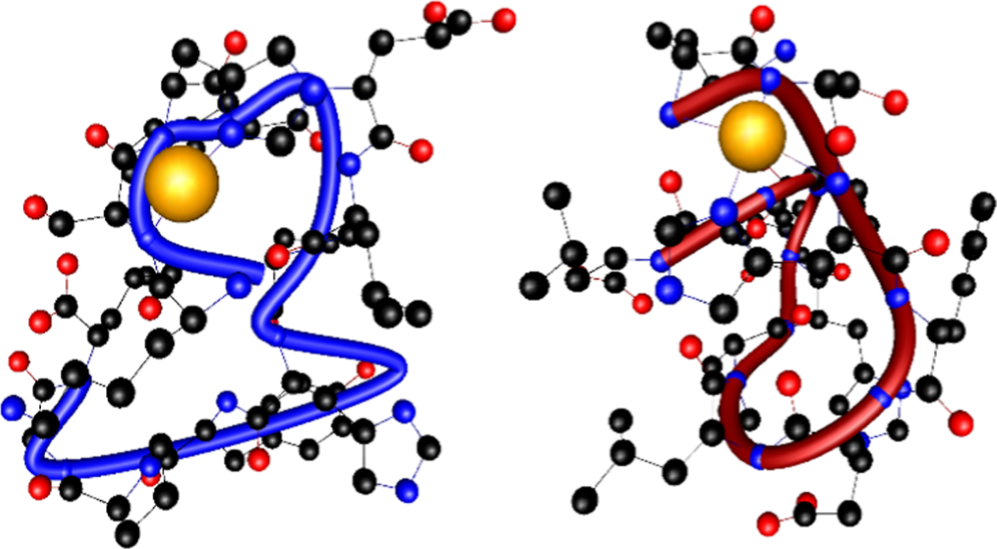
Structure of complexes with Cu(II) cation. The Cu(II)–L1 (KSHFELPHYPGL) on the left, the Cu(II)–L2 (kshfelphypgl) complex on the right. The tubes follow backbones.

To better understand this phenomenon (or, in other words, to solve this “puzzle”), computational studies were carried out using DFT. At the DFT level of theory, we found two multiple connected complexes with Cu(II) in the case of the L1 and L2 ligands, both containing the ATCUN motif (Figure 6).

Interestingly, the imidazole ring of the H8 residue in the Cu(II)–L2 complex forms a hydrogen bond with the carboxylate group (COO^–^) of E5 (H···O 1.733 Å, N···O 2.700 Å, N–H···O 152.6°), which, in turn, forms a hydrogen bond interaction with the N–H group of L6 *via* the second oxygen atom (H···O 1.608 Å, N···O 2.634 Å, and N–H···O 163.1°), as shown in Figure S9. We hypothesize that the cooperative nature of these interactions may contribute to the increased difficulty of H8 imidazole deprotonation, favoring deprotonation at the H3 imidazole (*N*_ε_) in the Cu(II)–L2 complex.

In conclusion, the quantum-chemistry methods show that both L- and d-amino acid-based ligands form thermodynamically stable, albumin-like complexes with Cu(II). The Cu(II)–L1 complex is more stable and forms shorter and stronger metal–ligand interactions. Interestingly, the H8 imidazole ring in the Cu(II)–L2 complex is involved in a strong, cooperative hydrogen-bonding interaction. Deprotonation of the H8 (*N*_δ_) imidazole in the Cu(II)–L2 complex is expected to require more energy compared to the Cu(II)–L1 complex. The increased difficulty of H8 imidazole deprotonation may favor deprotonation at the H3 (*N*_ε_) imidazole in the Cu(II)–L2 complex.

### Cu(II)–L3 (lgpyhplefhsk) System

L3 as a *retro-inverso* peptide has an inverted sequence and chirality compared to the native peptide L1, while maintaining an identical arrangement of side chains, which is also associated with the “loss” of the characteristic albumin-like binding motif, which is now located at the C-terminal part (in a reversed way)—L3 has the same chirality as L2 but an inverted sequence and a different arrangement of side chains (Figure 2).

Given the limited data available in the literature regarding the interaction of metal ions with RI peptides, a comprehensive analysis of the studied peptidomimetic will enable us to determine how this modification influences the coordination mode of Cu(II) ions and assesses the efficiency of Cu(II) ion binding in comparison with the native peptide.

L3 peptide starts to bind Cu(II) ions at a pH value similar to that of peptides L1 and L2. The first complex species observed is [CuH_3_L]^2+^, which reaches its maximum concentration at pH 4.6 (Figure 3C), and is most likely associated with the involvement of one or two histidine residues in Cu(II) coordination. The coordination of the imidazole nitrogen atom is confirmed by a band in the CD spectrum at 245 nm (Figure 5C); however, the precise determination of the coordination mode is difficult due to the almost complete overlap of this form with the subsequent complex species [CuH_2_L]^+^ (Figure 3C). The UV–vis spectrum with a maximum absorbance at 627 nm, obtained at a pH where the [CuH_2_L]^+^ complex reaches its maximum concentration, suggests a 2 N coordination. However, this does not exclude the presence of two complexes in equilibrium, one with two nitrogen atoms and the other with three nitrogen atoms as ligands, and this hypothesis is confirmed by the EPR spectra parameters (Figure S7C). The decrease in the p*K*_a_ value from 7.81 to 4.73 and the appearance of a new band at 275 nm in the CD spectrum at pH 6 may suggest coordination of the amino group from the N-terminus of the peptide. The next complex species, [CuHL], which begins to form at pH around 5 and reaches its maximum concentration at pH 8, is most likely formed as a result of coordination of the amide nitrogen atom, as evidenced by the characteristic bands in the CD spectrum: (i) at 628 nm and (ii) at 313 nm (Figure 5C). It is worth noting that the LMCT bands: for NH_2_ → Cu(II) (at 275 nm) and N_am_ → Cu(II) (at 313) appear simultaneously, which may indicate the coexistence in solution of complexes with {2N_im_, 1NH_2_} and {1N_im_, 1NH_2_, 1N_am_} donor sets.^69^ The formation of the next complex species, [CuL]^−^, is associated only with the deprotonation of the nonbinding Tyr residue (p*K*_a_ = 9.42 in the complex and p*K*_a_ = 9.72 in the free ligand). The parameters in UV–vis (ε_max_ at λ = 612 nm, Figure 4C) and EPR (*A*_∥_ = 160 G, *g*_∥_ = 2.23, Figure S7C) spectra indicate a typical 3 N coordination mode in the case of the [CuL]^−^ complex species. For the [CuH_–1_L]^2–^ complex, which reaches maximum concentration at pH 10.5, it remains unclear whether the Cu(II) ion is coordinated by three or four nitrogen atoms, or if an equilibrium between two distinct complexes exists with (i) {1N_im_, 1NH_2_, 1N_am_} and (ii) {1N_im_, 1NH_2_, 2N_am_} donor sets. This is suggested by both the broadened UV–vis bands and the EPR spectra, Figures 4C and S7C, respectively. At pH around 11, where the [CuH_–2_L]^3–^ species dominates, deprotonation of the Lys residue occurs and the coordination mode remains the same. Finally, at around pH 12.0, Cu(II) is clearly coordinated by four nitrogen atoms, resulting in (i) {1N_im_, 3N_am_} or (ii) {1NH_2_, 3N_am_} donors sets ([CuH_–3_L]^4–^ complex species). The disappearance of the band at 275 nm in the CD spectrum [characteristic of the NH_2_ → Cu(II) charge-transfer transition] suggests that the amino group in the coordination sphere of the Cu(II) ion has been replaced by an additional nitrogen atom from the peptide bond. 4 N coordination is supported by an UV–vis band at 521 nm (Figure 4C) and by characteristic CD bands for square-planar geometry, with (i) positive at 490 nm and (ii) negative at 610 nm Cotton effects (Figure 5C).

It is worth emphasizing that the coordination of the first amide nitrogen atom in the Cu(II)–L3 system and the formation of the 4 N complex with a square-planar geometry occur at a higher pH than that in the complexes of l-peptides with Cu(II) ions found in literature—in the complexes with the ATCUN motif at pH around 4–5 (as shown in this work and others^30,70,71^) and in the other ones usually around pH 7–8.^46,48,72−75^

To confirm the coordination mode, NMR studies were also performed at selected pH values, 5.4 and 7.4, which are important for the pH in the oral cavity.^76−79^

Before analyzing the metal ion influence, proton resonances of the peptide were assigned using conventional 2D ^1^H–^1^H TOCSY and NOESY experiments. As expected, at physiological pH, the majority of NH signals became highly broadened, and no significant correlations were detected in the NOESY spectra, suggesting the absence of a defined structure of L3 in aqueous solution, similar to what is typically observed for flexible peptides.^80^ Upon addition of Cu(II), broadening of selective NMR signals was observed at both acidic and physiological pH. The most affected residues are the ones belonging to His5 and His10 residues (Figure S10), suggesting their involvement in the metal coordination sphere. In fact, the copper-induced line broadenings are dependent on the dipolar coupling between the unpaired electron of the cupric ion and the nuclei close to the paramagnetic center.^81−83^ Besides His variations, the analysis of the ^1^H–^1^H TOCSY spectra also revealed the disappearance of Leu1 and Pro3 correlations in the presence of 0.2 and 0.3 Cu(II) equivalents at pH 7.4 only (Figure S11), indicating the participation of the N-terminal amino group in copper-ion binding, which is in good agreement with the suggestion described above for the [CuH_2_L]^+^ complex species.

### Copper-Binding Efficiency

**Figure 7:**
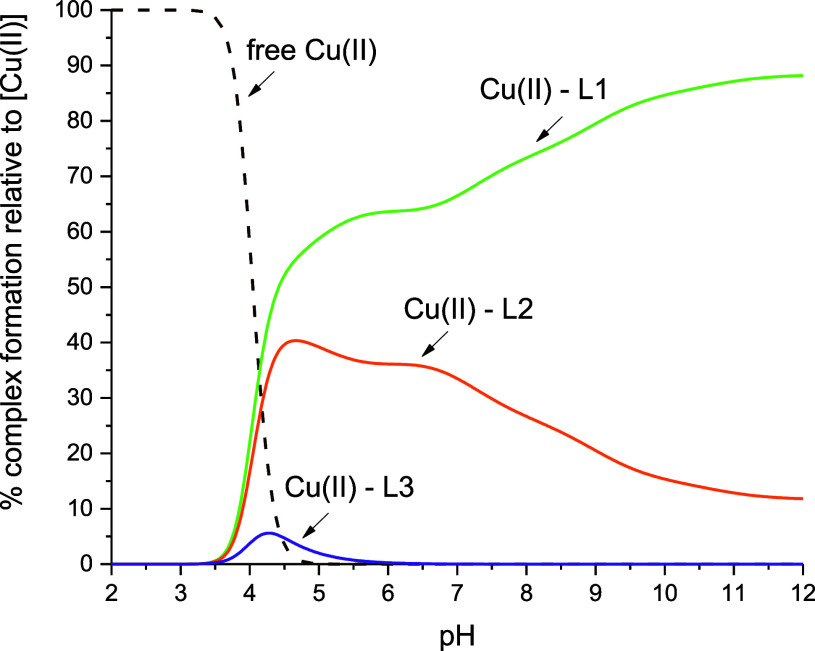
Competition plot between L1 (KSHFELPHYPGL), L2 (kshfelphypgl), and L3 (lgpyhplefhsk) with Cu(II) ions describing complex formation at different pH values in a hypothetical situation, in which equimolar amounts of the all reagents are mixed. Conditions: *T* = 25 °C, [Cu(II)] = [L1] = [L2] = [L3] = 0.001 M.

Comparing the efficiency of Cu(II) ion binding by the native peptide and its d-amino acid and *retro-inverso* analogues using the so-called competition plot [describing complex formation at different pH values in a hypothetical situation, in which equimolar amounts of all the reagents are mixed (Figure 7)], it is evident that the *retro-inverso* peptide has the lowest affinity compared to the ligands containing the ATCUN motif.

Interestingly, the competition plot clearly shows the difference in the Cu(II) ion-binding efficiency between ligands L1 and L2, which is perfectly explained by the tendency revealed by DFT studies—the formation of 4 N ATCUN-type coordination with the Cu(II) ion by both ligands was confirmed, and greater relative stability (Δ*E* = −65.30 kcal/mol) as well as shorter metal–ligand distances in the Cu(II)–L1 complex (2.152 Å for Cu(II)–L1 and 2.244 Å for Cu(II)–L2, Table 2) were observed.

The longer coordination bonds in the ATCUN motif of the Cu(II)–L2 system, and consequently the less stable complex, may result from an additional hydrogen bond between glutamic acid and the imidazole ring of the His(H8) residue (as described in the previous paragraph), which “prevents” the deprotonation of the His (H8) residue. This effect is reflected in the competition plot (Figure 7) by a much greater decrease in the binding affinity of the Cu(II) ion to the d-analogue (L2) mainly above pH 6.5 compared to the L1 peptide (in the Cu(II)–L1 system at pH 6.5, the His residue is already deprotonated).

### Influence of Metal Ion Coordination on the Secondary Structure and Antimicrobial Activity of Muc7 Fragments

Literature data indicate that changing the configuration of amino acids in a peptide sequence from l to d not only confers resistance to proteolysis (d-amino acids cannot be recognized by common proteases of the body and will not be easily degraded, especially in the case of the presence of Lys and Arg residues in the middle of the peptide sequence)^84^ but also may positively influence the formation of specific secondary structures and enhance antimicrobial potential.^26,85,86^ However, the literature shows that in some cases, the *retro-inverso* strategy does not always result in enhanced antimicrobial activity, as reported in a study by Neubauer and colleagues.^87^

### Secondary Structure Studies—Far-UV CD Spectroscopy

In the far-UV CD spectra of the studied systems, no significant changes were observed. As shown in Figure S12, in most cases, the obtained spectra (and their mirror images for spectra with ligands containing d-amino acids) are similar to those of a random coil, and the addition of Cu(II) ions does not significantly alter them. Therefore, it can be concluded that neither the substitution of l-amino acids with d-amino acids and the application of the *retro-inverso* strategy nor the coordination of Cu(II) induce significant changes in the secondary structures of the complexes with the ligands studied in this work, neither at pH 5.4 nor at pH 7.4.

### Antimicrobial Activity

The search for peptides as potential therapeutic agents against bacterial and fungal infections in oral diseases has gained significant attention in recent years. Oral cavity infections caused by pathogenic microorganisms can lead to serious conditions including dental caries, periodontitis, and oral candidiasis. AMPs have emerged as promising candidates due to their broad-spectrum activity and ability to disrupt biofilm formation. Several studies have identified specific peptides that exhibit strong activity against *S. mutans*, a major pathogen in dental caries, and *C. albicans*, responsible for oral candidiasis.^88−91^

To assess the antimicrobial efficacy of the MUC7 fragment, KSHFELPHYPGL, its peptidomimetics, and its corresponding Cu(II) complexes, a broth microdilution assay was employed. This technique enabled the determination of the MIC, defined as the lowest concentration at which the growth of the tested microbial species was effectively inhibited. Given the typically slightly acidic nature of saliva, with pH values ranging from 5.0 to 8.0, which fluctuate based on individual health conditions,^92^ we investigated the antimicrobial activity of the peptides and their copper(II) complexes against six bacterial species and *C. albicans*. The experiments were conducted at pH values of 7.4 (Table 3) and 5.4 (Table 4) to reflect physiological and mildly acidic conditions, respectively, which are both likely to occur in the human oral cavity. The antimicrobial properties of the studied ligands are significantly influenced by both pH levels and the presence of Cu(II) ions. At both tested pH conditions (Tables 3 and 4), the peptides L1 (KSHFELPHYPGL), L2 (kshfelphypgl), and L3 (lgpyhplefhsk) exhibited enhanced antimicrobial activity upon Cu(II) coordination. As demonstrated in Tables 3 and 4, all peptides and their corresponding metal complexes were more effective against Gram-positive bacteria than against Gram- negative ones. Among the tested bacterial strains, *S. mutans* was the most susceptible. At pH 5.4, the MICs for *S. mutans* were 125 μg/mL for L1 and 250 μg/mL for both L2 and L3. Although the activity against Gram-negative bacteria (*E. coli* and *P. aeruginosa*) is lower, it is noteworthy that peptidomimetics exhibit greater antimicrobial potency in complexes with Cu(II) ions compared to the native peptide (Tables 3 and 4).

**Table 3:** Antibacterial and Anti-*Candida* Activities of Peptides/Complexes Were Assessed *In Vitro* by Determining Their MICs (μg/mL)^a^

strain	KSHFELPHYPGL (L1)		kshfelphypgl (L2)		lgpyhplefhsk (L3)	
	L1	+Cu(II)	L2	+Cu(II)	L3	+Cu(II)
*E. coli*	*n*/*d*	*n*/*d*	*n*/*d*	500	*n*/*d*	*n*/*d*
ATCC 25922						
*P. aeruginosa*	*n*/*d*	*n*/*d*	*n*/*d*	500	*n*/*d*	500
ATCC 15422						
*S. aureus*	*n*/*d*	*n*/*d*	500	500	*n*/*d*	*n*/*d*
ATCC 259 23						
*E. faecalis*	*n*/*d*	*n*/*d*	*n*/*d*	*n*/*d*	*n*/*d*	500
ATCC 29212						
*S. mutans*	125	125	500	250	500	250
PCM 2502						
*S. sanguinis*	500	250	500	250	500	500
PCM 2335						
*C. albicans*	250	125	*n*/*d*	250	500	500
SC5314						

a Antimicrobial tests were conducted in a 10 mM HEPES buffer at pH 7.4. Experiments were performed for peptides and their copper(II) complexes. *n*/*d*, not determined within the concentration range used in this study.

**Table 4:** Antibacterial and Anti-*Candida* Activities of Peptides/Complexes Were Assessed *In Vitro* by Determining Their MICs (μg/mL)^a^

strain	KSHFELPHYPGL (L1)		kshfelphypgl (L2)		lgpyhplefhsk (L3)	
	L1	+Cu(II)	L2	+Cu(II)	L3	+Cu(II)
*E. coli*	*n*/*d*	*n*/*d*	*n*/*d*	500	*n*/*d*	*n*/*d*
ATCC 25922						
*P. aeruginosa*	*n*/*d*	500	500	125	*n*/*d*	250
ATCC 15422						
*S. aureus*	*n*/*d*	500	250	125	*n*/*d*	500
ATCC 25923						
*E. faecalis*	*n*/*d*	*n*/*d*	*n*/*d*	500	*n*/*d*	500
ATCC 29212						
*S. mutans*	125	62.5	250	125	250	125
PCM 2502						
*S. sanguinis*	250	125	500	250	500	250
PCM 2335						
*C. albicans*	125	62.5	250	125	250	250
SC5314						

a Antimicrobial tests were conducted in a 10 mM MES buffer at pH 5.4. Experiments were performed for peptides and their copper(II) complexes. *n*/*d*, not determined within the concentration range used in this study.

Interestingly, Cu(II) coordination and slight acidic pH increased the antimicrobial efficacy against *S. mutans* of all the studied ligands, with the Cu(II)–L1 complex showing the lowest MIC of 62.5 μg/mL (Table 4). Furthermore, the peptides L1, L2, and L3 also display intrinsic antifungal activity against *Candida* species and, analogously to the case of *S. mutans*, Cu(II) binding caused an at least two-fold increase in antimicrobial activity in case of L1 and L2.

The findings indicated that Cu(II) ions (without peptides) did not exhibit antibacterial activity against Gram-positive and Gram-negative bacterial strains nor against *C. albicans* at the concentrations applied in the peptide complexes, ranging from 0.2 to 22 μg/mL (data not shown).

Peptidomimetics designed to replicate the functional domains of MUC7 show significant antimicrobial activity against bacterial and fungal pathogens, particularly in the context of a rising antimicrobial resistance. Notably, peptides L1, L2, and L3 exhibited enhanced antimicrobial properties against *S. mutans* and various *Candida* species after Cu(II) binding. Our findings indicate that the coordination of this metal ion may be one of the ways in which nature modulates the antimicrobial efficacy of these AMPs. We hypothesize that the studied ligands in complexes with Cu(II) ions may lead, among others, to the destabilization or permeabilization of the cell wall—a phenomenon supported by prior studies—which, in this case, could be attributed to Cu(II)-ATCUN region-mediated ROS generation.^93,94^

Peptidomimetics have attracted significant attention in recent years due to their potential as antimicrobial agents, particularly in the context of rising antibiotic resistance. These peptides, capable of forming stable complexes with metal ions, possess the ability to transport cations across cellular membranes, disrupting the ionic balance of microbial cells. The mechanism of action of peptide ionophores is primarily based on their ability to bind to metal ions, such as Cu(II), Zn(II), or Ca(II), and facilitate their transport across lipid bilayers, often leading to ion imbalance and cell death.^94^

Metal complexes formed with known AMPs frequently exhibit distinct mechanisms of action compared with individual peptides. These include the disruption of bacterial cell membranes and the metal-ion-mediated hydrolytic or oxidative degradation of nucleic acids.^93^ Such metal-based compounds have gained considerable attention due to their wide-ranging potential applications. Gram-negative bacteria, with their outer membrane rich in lipopolysaccharides, present a more challenging target for metal-based antibiotics than Gram-positive bacteria, whose peptidoglycan-rich cell walls are more accessible.^93^ Therefore, the interaction between metal ions and peptide structures is a crucial factor in the antimicrobial efficacy of these compounds.

## Conclusions

The interactions of d-amino-acid-based AMPs with Cu(II) ions represent a novel and intriguing area of bioinorganic chemistry. Our findings confirm that both the native peptide (L1) and its d-amino acid analogue (L2) coordinate Cu(II) *via* the ATCUN motif, but with notable differences in stability. In L2, a cooperative hydrogen bond between Glu and His-8 prevents His-8 deprotonation while promoting His-3 deprotonation, leading to reduced complex stability. This decreased stability correlates with slightly diminished antimicrobial activity (higher MIC values), particularly at an acidic pH (5.4). In contrast, the native MUC7 fragment formed the most potent Cu(II) complex (MIC = 62.5 μg/mL against *S. mutans* and *C. albicans* at pH 5.4), underscoring the importance of the native ATCUN motif in metal-mediated activity. For the first time, we report Cu(II) complexes with *retro-inverso* (RI) peptides, which lack the ATCUN motif and exhibit a distinct coordination mode. A particularly interesting feature is the delayed involvement of amide residues in coordination, observed at a more basic pH than expected for l-amino acid systems.

Our results provide valuable insights into the bioinorganic chemistry of antimicrobial peptidomimetics, highlighting their potential in combating antibiotic resistance while addressing challenges like proteolytic stability. Further research is essential to unraveling their complex chemistry and expanding their therapeutic applications.

## Supporting Information Available

Certificates of analysis of synthesized peptides; thermodynamic and spectroscopic data for proton of MUC7 fragment and its peptidomimetics; potentiometric titration curves for MUC7 fragment and its peptidomimetic and its Cu(II) complexes; mass spectra for the metal-peptide systems; EPR spectra for Cu(II)—MUC7 fragment and its peptidomimetics; structure of hydrogen-bond interactions in L2; ^1^H NMR Cu(II) titrations of the L3 system at pH 5.4 and 7.4; NMR ^1^H–^1^H TOCSY spectra of L3 with 0.2 and 0.3 Cu(II) eqs.; and far-UV CD spectra for MUC7 fragment, its peptidomimetics, and its metal complexes (PDF)

## Abbreviations

AMPs: antimicrobial peptides
ATCC: American-type culture collection
ATCUN: amino-terminal Cu(II)- and Ni(II)-binding site
BHI: brain heart infusion
CD: circular dichroism
DFT: density functional theory
EPR: electron paramagnetic resonance
LPS: lipopolysaccharides
MIC: minimal inhibitory concentration
MDR: multidrug-resistant
MHB: Mueller–Hinton broth
MUC7: Mucin 7
NMR: nuclear magnetic resonance
PCM: Polish Collection of Microorganisms
RI: *retro-inverso*
YPD: yeast peptone dextrose.
