# Supplementary material for: Modulating Copper(II) Coordination and Antimicrobial Activity: Effects of d-Amino Acid Substitution and Retro-Inverso Modification in Human Saliva MUC7 Peptide
Source: Inorg Chem. 2025 Mar 19;64(12):6365–77. doi: 10.1021/acs.inorgchem.5c00438 (PMC11962837; doi:10.1021/acs.inorgchem.5c00438)
Supplement: Supplementary file 1 — ic5c00438_si_001.pdf [file ic5c00438_si_001.pdf]

Supplementary Information for

Modulating Copper(II) Coordination and Antimicrobial Activity: Effects of D-Amino Acid Substitution and *Retro-Inverso* Modification in Human Saliva MUC7 Peptide

Joanna Wątły,[*a] Klaudia Szarszoń, [a] Monika Sabieraj, [a] Arian Kola, [b, c] Robert Wieczorek, [a] Tomasz Janek [d] and Daniela Valensin [b, e]*

[a] Faculty of Chemistry, University of Wrocław, F. Joliot-Curie 14, 50-383 Wrocław, Poland. *joanna.watly2@uwr.edu.pl

[b] Department of Biotechnology, Chemistry and Pharmacy, University of Siena, Via A. Moro 2, 53100 Siena, Italy

[c] Department Life Science, University of Siena, Via A. Moro 2, 53100 Siena, Italy

[d] Department of Biotechnology and Food Microbiology, Wrocław University of Environmental and Life Sciences, Chełmońskiego 37, 51-630, Wrocław, Poland

[e] CIRMMP, Via Luigi Sacconi 6, 50019 Firenze, Italy

Table of Contents

Figure S 1. Certificates of analysis of synthesized peptides: A) KSHFELPHYPGL (L1, L-amino acid peptide, native peptide), B) kshfelphypgl (L2, D-amino acid analogue), and C) lgpyhplefhsk (L3, peptide synthesized by *retro-inverso* strategy, RI). S11

Figure S 2. Representative distribution diagram for (A) L1 (KSHFELPHYPGL); (B) L2 (kshfelphypgl) and (C) L3 (lgpyhplefhsk) peptides in aqueous solution of 4 mM $HClO_4$ with I = 0.1 M $NaClO_4$ dependent on pH values. C_L = 0.4 mM; molar ratio M:L – 0.8:1........ S12

Figure S 3. The comparison of the potentiometric titration curves shape for the native peptide (L1, L-aa, black line) and D-amino acids analogue (L2, D-aa, red dotted line)........ S13

Figure S 4. ESI-MS spectra of Cu(II) complexes with KSHFELPHYPGL (L1) peptide for chosen m/z region (A). Comparisons of experimental (red) and simulated (gray) spectra are presented for selected signals (B) and (C). Molar ratio M:L – 1:1. [L] = 0.0001 M. Samples prepared in MeOH:H_2O (50:50) mixture. S14

Figure S 5. ESI-MS spectra of Cu(II) complexes with kshfelphypgl (L2) peptide for chosen m/z region (A). Comparisons of experimental (red) and simulated (gray) spectra are presented for selected signals (B). Molar ratio M:L – 1:1. [L] = 0.0001 M. Samples prepared in MeOH:H_2O (50:50) mixture....... S15
Figure S 6. ESI-MS spectra of Cu(II) complexes with lgpyhplefhsk (L3) peptide for chosen m/z region (A). Comparisons of experimental (red) and simulated (gray) spectra are presented for selected signals (B). Molar ratio M:L – 1:1. [L] = 0.0001 M. Samples prepared in MeOH:H_2O (50:50) mixture....... S16
Figure S 7. pH-dependent EPR spectra for (A) Cu(II) – L1 (KSHFELPHYPGL); (B) Cu(II) – L2 (kshfelphypgl) and (C) Cu(II) – L3 (lgpyhplefhsk) systems in aqueous solution with the addition of ethylene glycol (30%) at temperature 77 K; [Cu(II)] = 0.001 M; molar ratio M:L equal to 0.8:1...... S17
Figure S 8. The comparison of the potentiometric titration curves shape for the Cu(II) complexes with native peptide (L1, L-aa, black line) and D-amino acids analogue (L2, D-aa, red dotted line). S18
Figure S 9. The structure of hydrogen-bond interactions between the backbone of E5, the side chain of L6, and the side chain of H8 residues... S18
Figure S 10. ^{1}H NMR Cu(II) titrations of the L3 system at pH 5.4 (left) and 7.4 (right). The effects induced by increasing amounts of Cu(II) are shown in the aromatic region of the NMR spectra. [L3]= 0.5 mM. T=298 K. MES-d_{13} 20 mM and phosphate buffer 20 mM. for pH 5.4 and 7.4. respectively.S19
Figure S 11. Comparison of NMR ^{1}H-^{1}H TOCSY spectra of L3 (lgpyhplefhsk) 0.5 mM alone (black contours). in presence of 0.2 Cu(II) eqs. (magenta contours) and in presence of 0.3 Cu(II) eqs. (blue contours). T =298 K. pH 7.4. 20 mM phosphate buffer. S19
Figure S 12. CD spectra in the far-UV (180-250 nm) region at chosen pH values: 5.4 and 7.4 for the Cu(II) complexes with (A) L1 (KSHFELPHYPGL),(B) L2 (kshfelphypgl) and (C) L3 (lgpyhplefhsk) ligands in aqueous solution of 4 mM $HClO_4$ with I = 0.1 M $NaClO_4$; molar ratio M:L 0.8:1; the optical path length = 0.2 mm; C_L = 0.3 mM; dashed lines correspond to the ligand spectra; dotted lines correspond to recorded spectra with opposite sign for better comparison with the native - L-amino acid peptide. S20

Table S 1. *m/z* values (for monoisotopic masses) for individual ions of the complex forms and ligands (with the highest intensity) obtained by ESI mass spectrometry at pH 6. .... S13

A

KareBay™ Biochem
Experts in Custom Reagents, Kits & Services

KareBay Biochem, Inc.

CERTIFICATION OF ANALYSIS

Product Information:

Product Name	
Cat. NO.	825742
Lot:	22112105
Size:	80mg
Sequence:	KS HFELP HYPGL
Molecular Weight:	1424.59
Storage:	-20℃

Analysis Summary:

Test Items	Standard	Result
HPLC Trace:	≧98.00%	98.33%
Mass Spectrometry:	Consistent	Consistent
Appearance:	White powder	White powder

Caution:
For laboratory or further manufacturing use only. Not for household or any human being related utilize. If there is any further question, please contact **KareBay Biochem, Inc.** at:
Tel: 732-823-1545
E-mail: support@karebaybio.com.

Address: 11 Deer Park Drive, Suite 102A, Monmouth Junction, NJ 08852
Telephone:732-823-1545 **Email:** service@karebaybio.com **Website:** www.karebaybio.com

825742 HPLC Analysis Report

Sequence:KS HFELP HYPGL
Sample ID:825742
Lot:22112105
Buffer A:0.1% TFA in 100% water(v/v)
Buffer B:0.1% TFA in 100% acetonitrile(v/v)
Gradient10-80% Buffer B in 20min
Flow:1ml/min Wavelength:220nm
Column: Agilent Pursuit 5um C18 4.6*250mm

Chromatogram

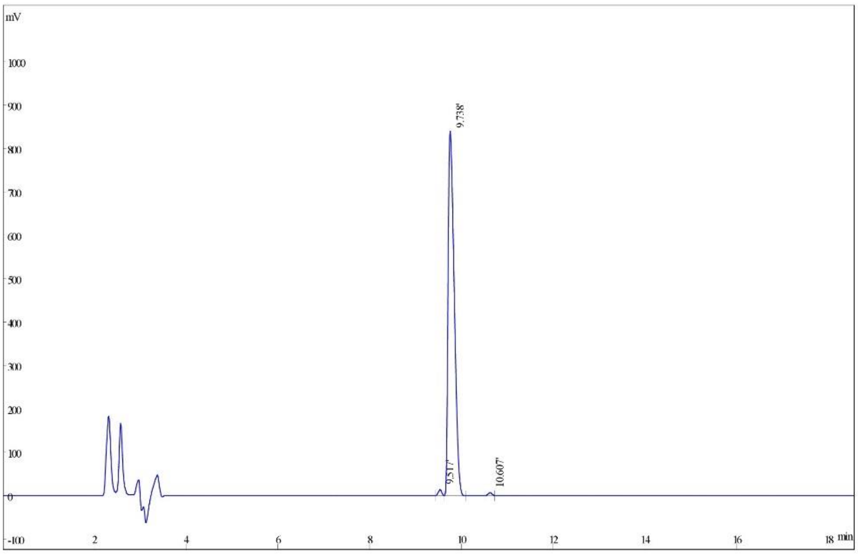

Rank	Time	Quantity	Area	Height
1	9.517	1.027	81729	14665
2	9.738	98.33	7824053	843002
3	10.607	0.6436	51211	7809
Total		100	7956993	865476

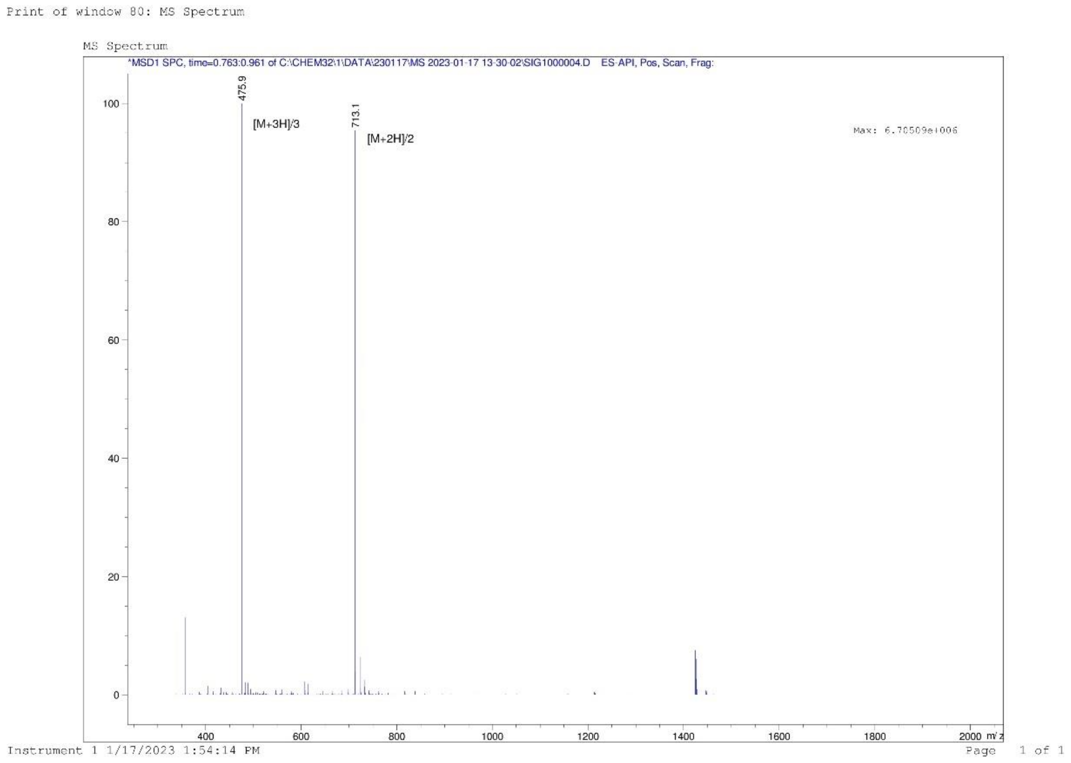
Print of window 80: MS Spectrum
MS Spectrum
*MSD1 SPC, time=0.763:0.961 of C:\CHEM32\1\DATA\230117\MS 2023-01-17 13-30-02\SIG1000004.D ES-API, Pos, Scan, Frag:
475.9
[M+3H]/3
713.1
[M+2H]/2
Max: 6.70509e+006
100
80
60
40
20
0
400
600
800
1000
1200
1400
1600
1800
2000
m/z
Instrument 1 1/17/2023 1:54:14 PM
Page 1 of 1

B

KareBay™ Biochem
Experts in Custom Reagents, Kits & Services

KareBay Biochem, Inc.

CERTIFICATION OF ANALYSIS

Product Information:

Product Name	
Cat. NO.	825744
Lot:	22112107
Size:	80mg
Sequence:	KSHFELPHYPGL (all D-amino acids)
Molecular Weight:	1424.60
Storage:	-20℃

Analysis Summary:

Test Items	Standard	Result
HPLC Trace:	≧98.00%	98.81%
Mass Spectrometry:	Consistent	Consistent
Appearance:	White powder	White powder

Caution:
For laboratory or further manufacturing use only. Not for household or any human being related utilize. If there is any further question, please contact **KareBay Biochem, Inc.** at:

Tel: 732-823-1545
E-mail: support@karebaybio.com.

825744 HPLC Analysis Report

Sequence:KSHFELPHYPGL (all D-amino acids)
Sample ID:825744
Lot:22112107
Buffer A:0.1% TFA in 100% water(v/v)
Buffer B:0.1% TFA in 100% acetonitrile(v/v)
Gradient10-80% Buffer B in 20min
Flow:1ml/min Wavelength:220nm
Column: Agilent Pursuit 5um C18 4.6*250mm

Chromatogram

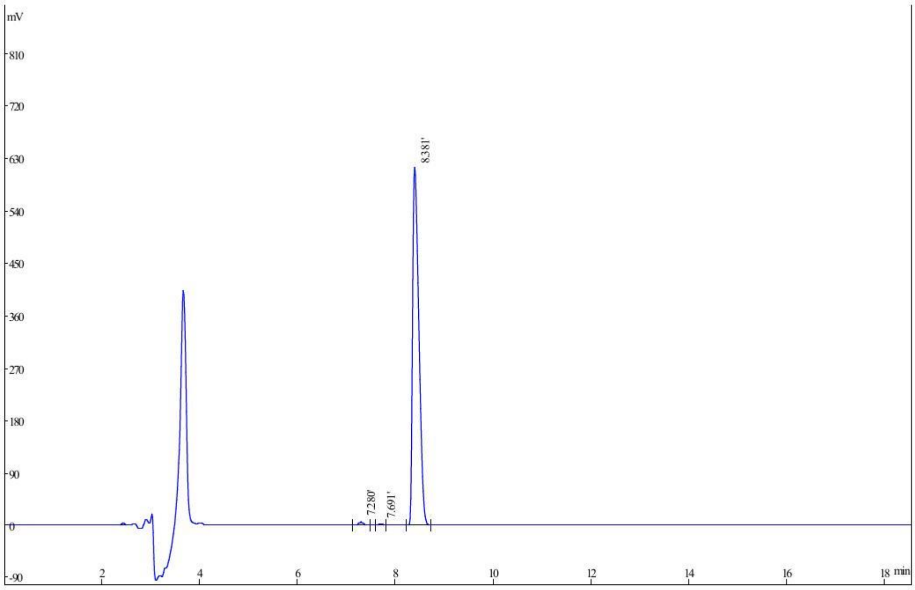

Rank	Time	Quantity	Area	Height
1	7.280	0.8012	44469	6184
2	7.691	0.3871	21486	3796
3	8.381	98.81	5484289	616440
Total		100	5550244	626420

Print of window 80: MS Spectrum

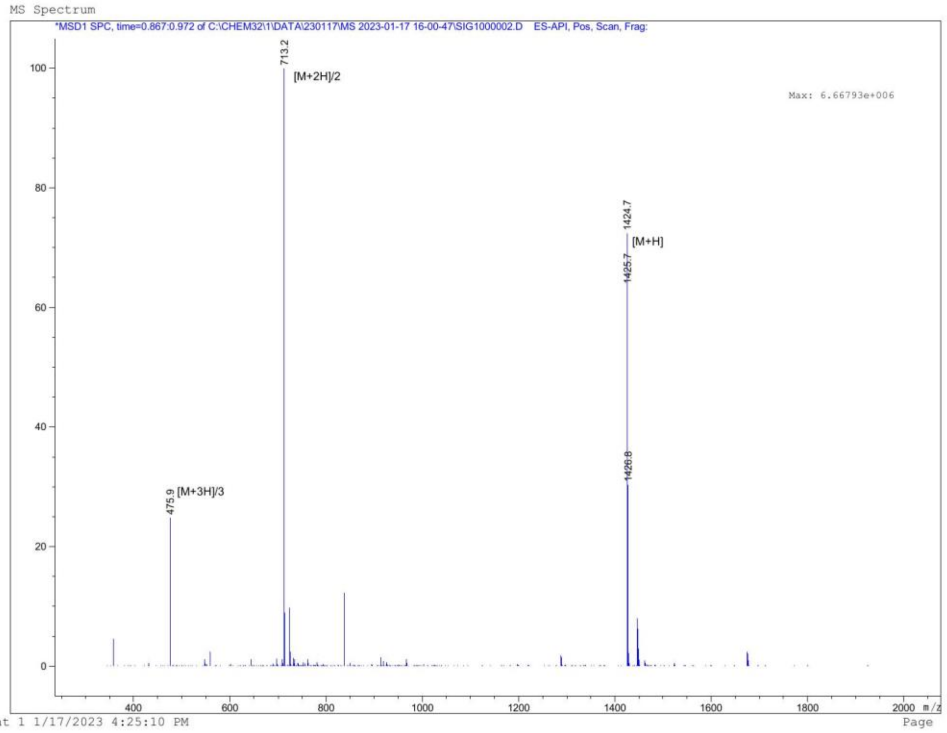

Instrument 1 1/17/2023 4:25:10 PM Page 1 of 1

C

KareBay™ Biochem
Experts in Custom Reagents, Kits & Services

KareBay Biochem, Inc.

CERTIFICATION OF ANALYSIS

Product Information:

Product Name	
Cat. NO.	825745
Lot:	22112108
Size:	80mg
Sequence:	LG PYHPL EFHSK (all D-amino acids)
Molecular Weight:	1424.6
Storage:	-20℃

Analysis Summary:

Test Items	Standard	Result
HPLC Trace:	≧98.00%	98.52%
Mass Spectrometry:	Consistent	Consistent
Appearance:	White powder	White powder

Caution:
For laboratory or further manufacturing use only. Not for household or any human being related utilize. If there is any further question, please contact **KareBay Biochem, Inc.** at:

Tel: 732-823-1545
E-mail: support@karebaybio.com.

Address: 11 Deer Park Drive, Suite 102A ,Monmouth Junction, NJ 08852
Telephone:732-823-1545 **Email:** service@karebaybio.com **Website:** www.karebaybio.com

825745 HPLC Analysis Report

Sequence:LG PYHPL EFHSK (all D-amino acids)
Sample ID:825745
Lot:22112108
Buffer A:0.1% TFA in 100% water(v/v)
Buffer B:0.1% TFA in 100% acetonitrile(v/v)
Gradient10-80% Buffer B in 20min
Flow:1ml/min Wavelength:220nm
Column: Agilent Pursuit 5um C18 4.6*250mm

Chromatogram

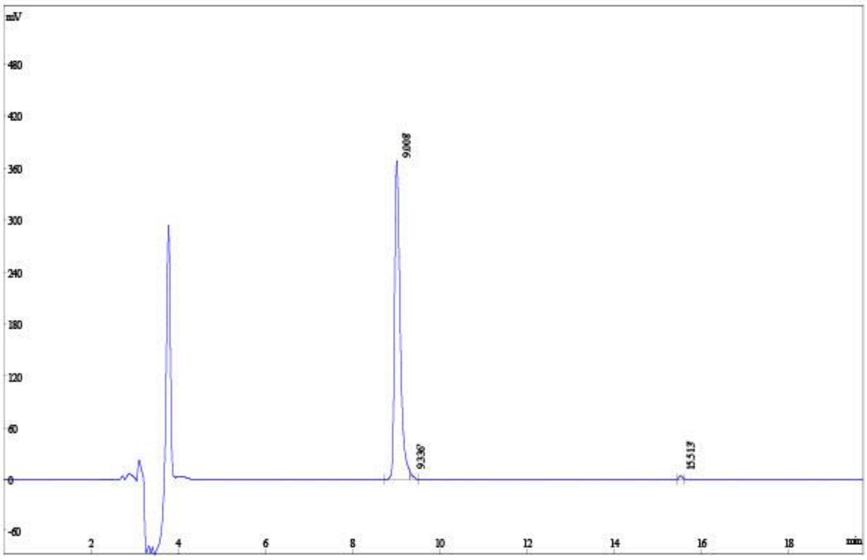

Rank	Time	Quantity	Area	Height
1	9.008	98.52	3313892	369606
2	9.336	0.769	25864	6373
3	15.513	0.707	23779	4128
Total		100	3363535	380107

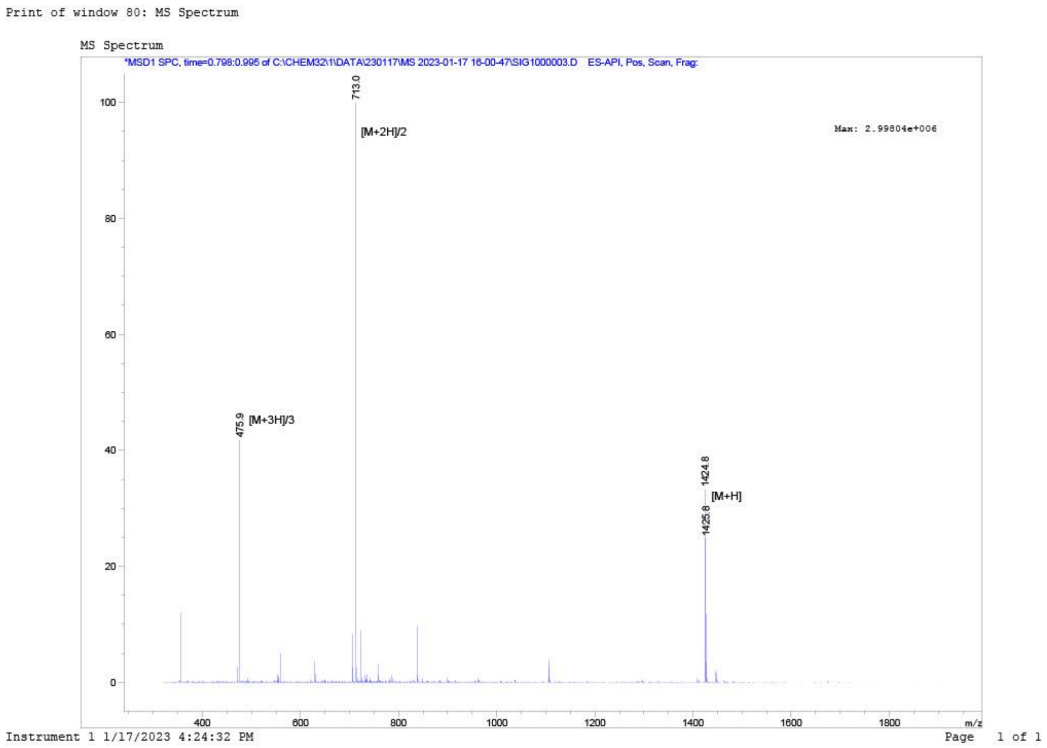

Figure S 1. Certificates of analysis of synthesized peptides: A) KSHFELPHYPGL (L1, L-amino acid peptide, native peptide), B) kshfelphypgl (L2, D-amino acid analogue), and C) lgpyhplefhsk (L3, peptide synthesized by retro-inverso strategy, RI).

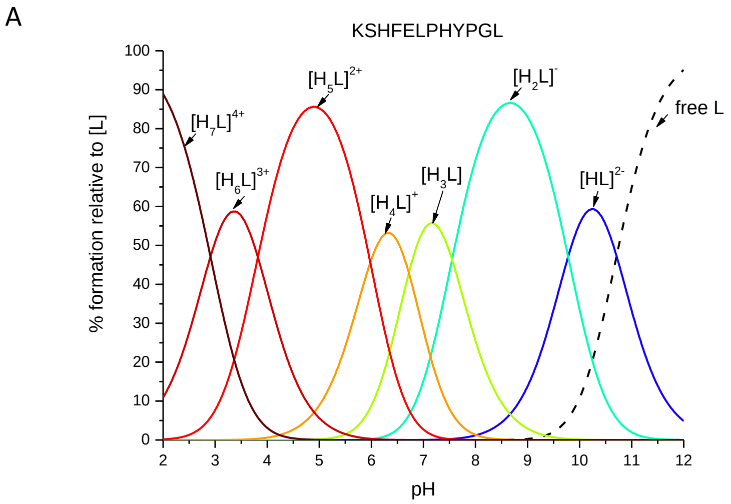

B

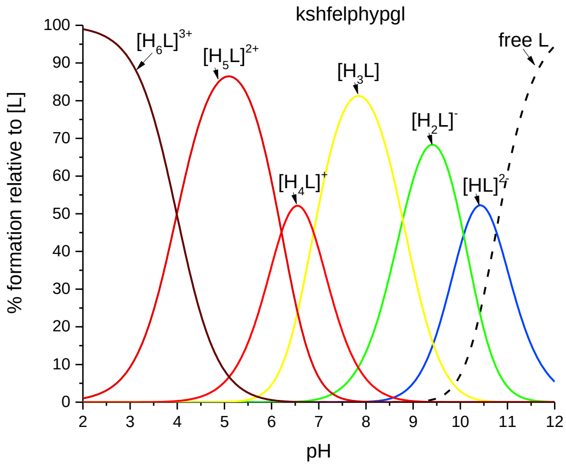

C

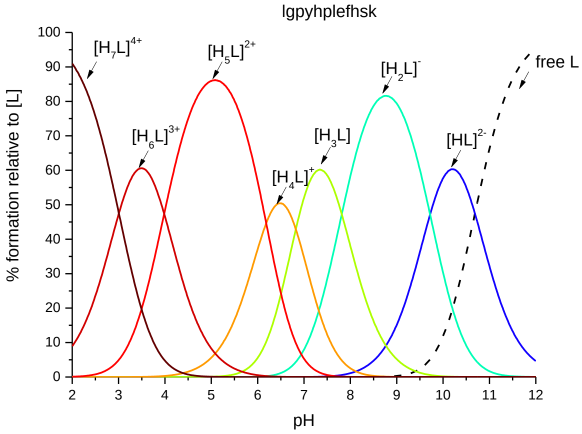

Figure S 2. Representative distribution diagram for (A) L1 (KSHFELPHYPGL); (B) L2 (kshfelphypgl) and (C) L3 (lgpyhplefhsk) peptides in aqueous solution of 4 mM $HClO_4$ with I = 0.1 M $NaClO_4$ dependent on pH values. C_L = 0.4 mM.

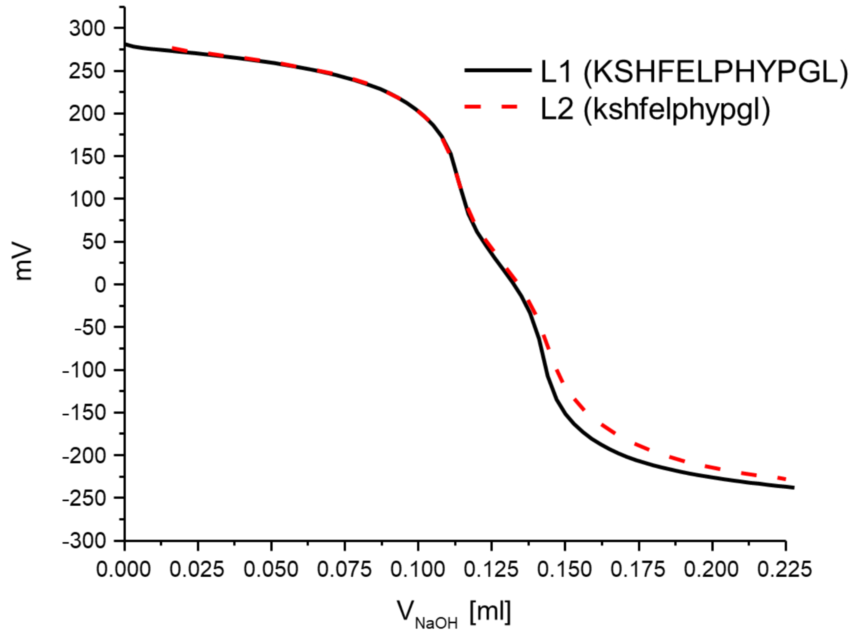

Figure S 3. The comparison of the potentiometric titration curves shape for the native peptide (L1, L-aa, black line) and D-amino acids analogue (L2, D-aa, red dotted line).

Table S 1. m/z values (for monoisotopic masses) for individual ions of the complex forms and ligands (with the highest intensity) obtained by ESI mass spectrometry at pH 6.

**KSHFELPHYPGL (L1)**	
**Form**	**Monoisotopic mass *m/z***
$[L]^{2+}$	712.8697
$[CuL]^{2+}$	743.3267
$[L]^{3+}$	475.5822
$[CuL]^{3+}$	495.8869
$[L+Na]^{3+}$	482.9095
$[CuL+K]^{3+}$	508.5388
$[CuL+Na]^{3+}$	503.2142
**kshfelphypgl (L2)**	
**Form**	**Monoisotopic mass *m/z***
$[L]^{2+}$	712.8697
$[CuL]^{2+}$	743.3267
$[L+Na+2K]^{2+}$	762.8244
**lgpyhplefhsk (L3)**	
**Form**	**Monoisotopic mass *m/z***
$[L]^{2+}$	712.8697
$[L+Na]^{2+}$	723.8607
$[L+Na+2K]^{2+}$	762.8244
$[CuL]^{2+}$	743.3267

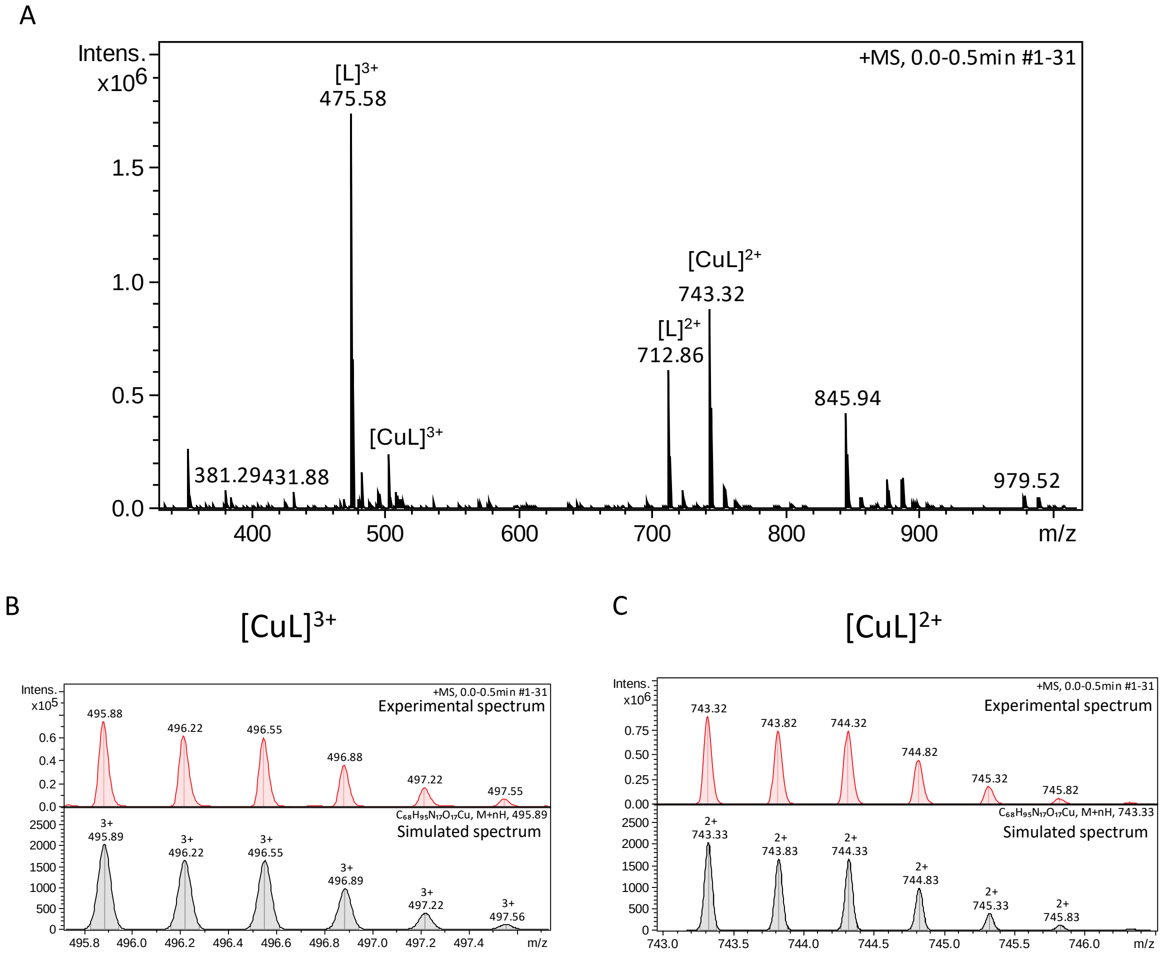

Figure S 4. ESI-MS spectra of Cu(II) complexes with KSHFELPHYPGL (L1) peptide for chosen m/z region (A). Comparisons of experimental (red) and simulated (gray) spectra are presented for selected signals (B) and (C). Molar ratio M:L – 1:1. [L] = 0.0001 M. Samples prepared in MeOH:H_2O (50:50) mixture.

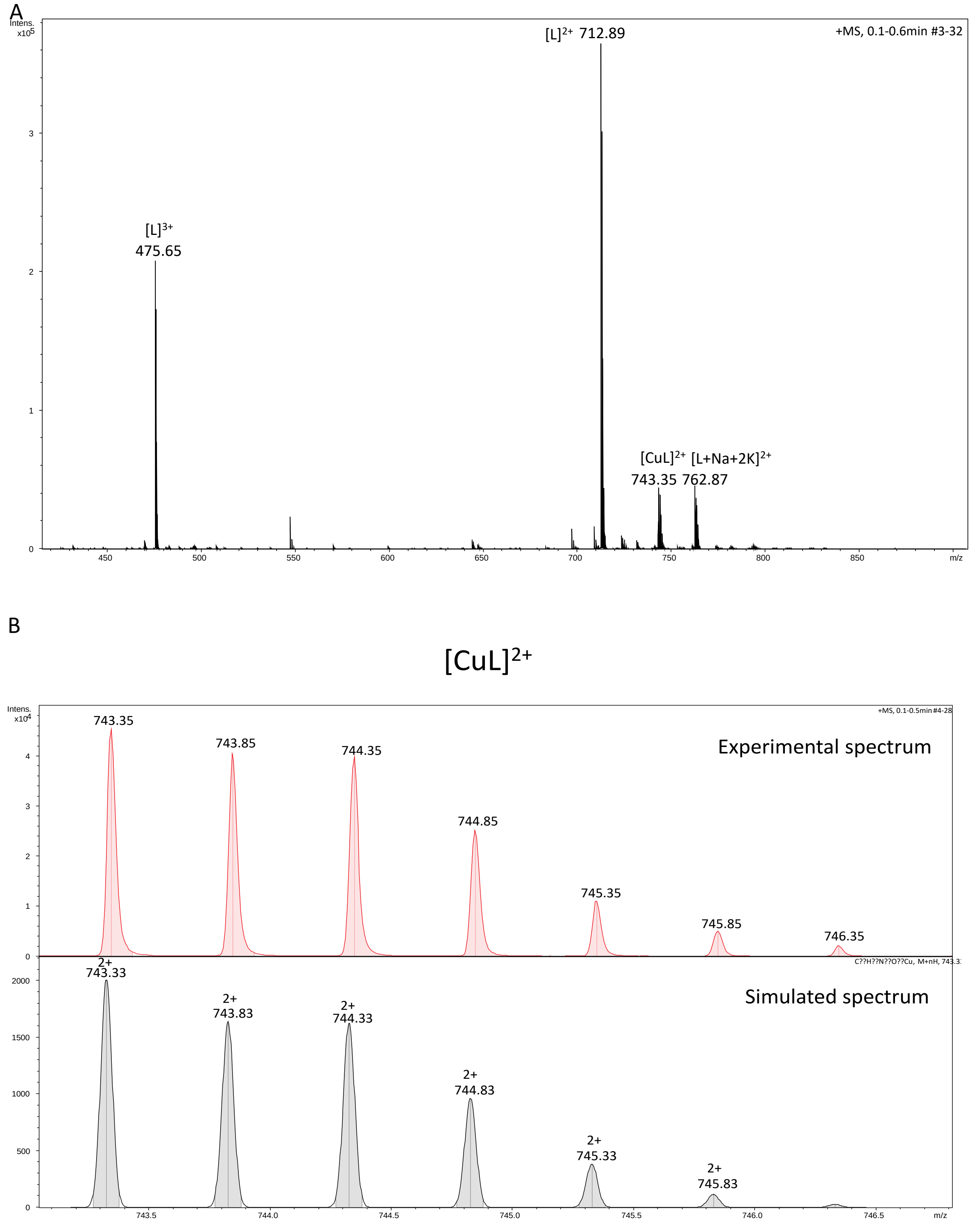

Figure S 5. ESI-MS spectra of Cu(II) complexes with kshfelphypgl (L2) peptide for chosen m/z region (A). Comparisons of experimental (red) and simulated (gray) spectra are presented for selected signals (B). Molar ratio M:L – 1:1. [L] = 0.0001 M. Samples prepared in MeOH:H_2O (50:50) mixture.

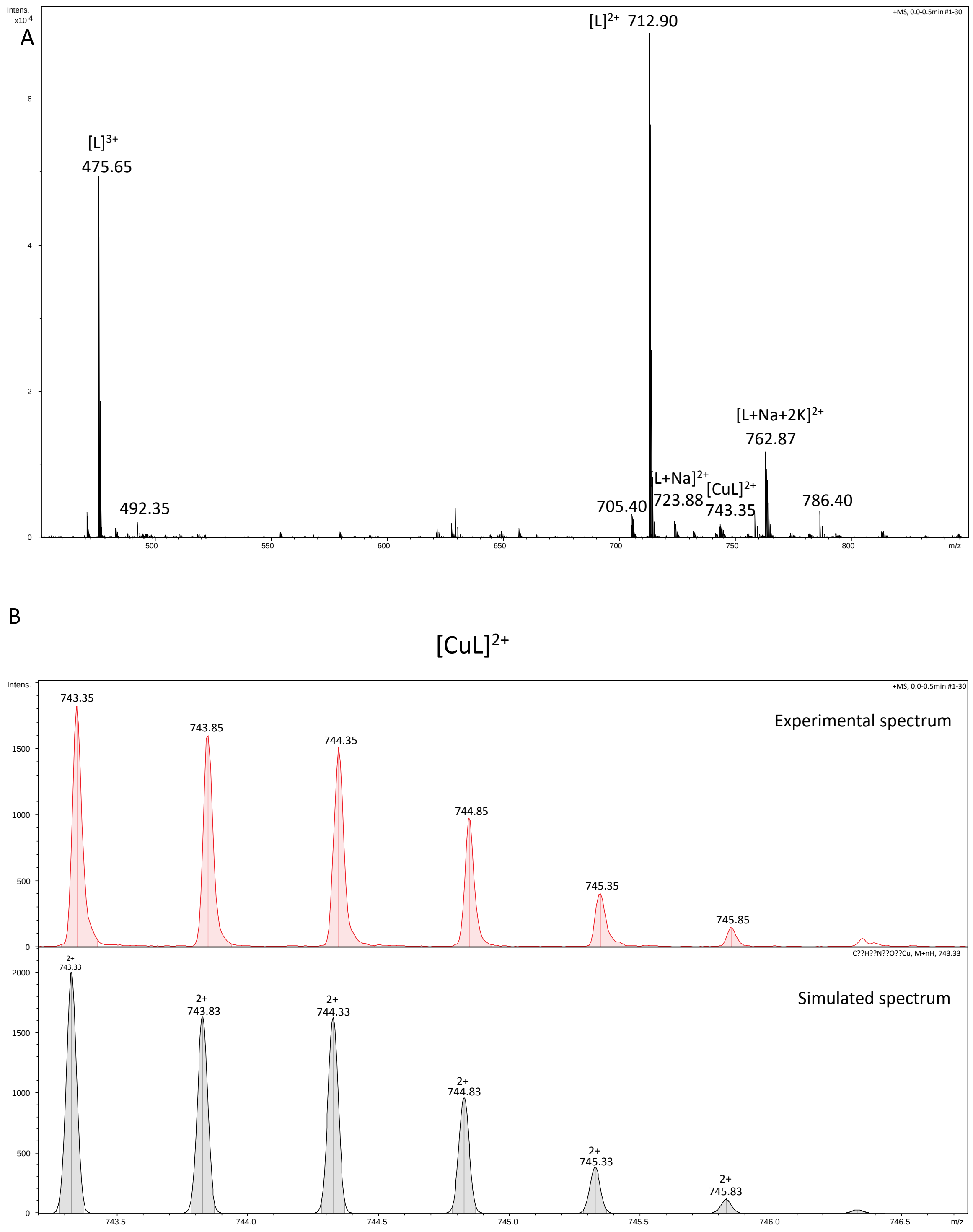

Figure S 6. ESI-MS spectra of Cu(II) complexes with lgpyhplefhsk (L3) peptide for chosen m/z region (A). Comparisons of experimental (red) and simulated (gray) spectra are presented for selected signals (B). Molar ratio M:L – 1:1. [L] = 0.0001 M. Samples prepared in MeOH:H_2O (50:50) mixture.

A

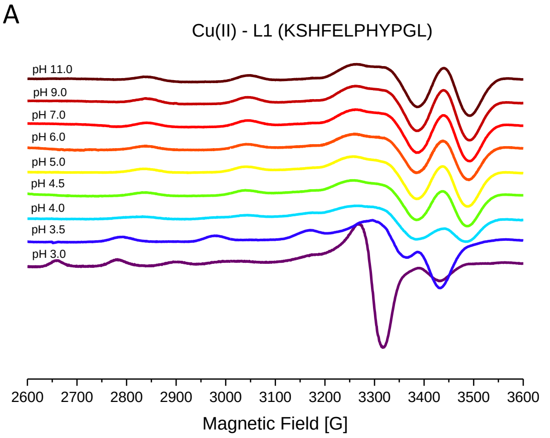

pH	$A_{\|\|}$ [G] (A_{zz})	$g_{\|\|}$ (g_{zz})	MW Frequency [GHz]	Suggested coordination mode
3.0	121.9	2.42	9.6001	4O
3.5	189.2	2.24	9.5985	3N
4.0	192.1<br>202.2	2.23<br>2.19	9.6010	3N + 4N
4.5	203.0	2.18	9.6003	4N
5.0	205.0	2.18	9.5991	4N
6.0	205.0	2.18	9.5968	4N
7.0	205.0	2.18	9.5996	4N
9.0	205.0	2.18	9.5985	4N
11.0	205.0	2.18	9.6006	4N

B

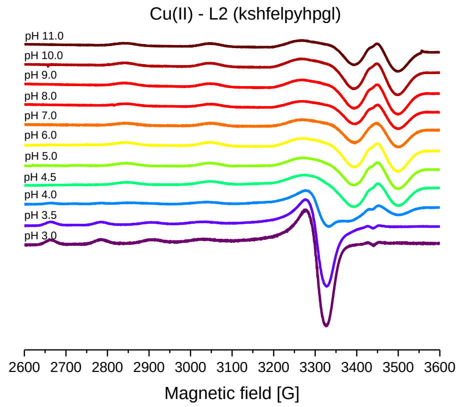

pH	$A_{\|\|}$ [G] (A_{zz})	$g_{\|\|}$ (g_{zz})	MW Frequency [GHz]	Suggested coordination mode
3.0	119.3	2.42	9.6320	4O
3.5	120.5	2.42	9.6308	4O
4.0	124.6<br>190.8	2.38<br>2.20	9.6295	4O + 4N
4.5	202.9	2.18	9.6289	4N
5.0	202.9	2.18	9.6274	4N
6.0	202.4	2.18	9.6266	4N
7.0	202.9	2.18	9.6290	4N
8.0	202.9	2.18	9.6283	4N
9.0	202.9	2.18	9.6260	4N
10.0	202.9	2.18	9.6240	4N
11.0	202.9	2.18	9.6262	4N

C

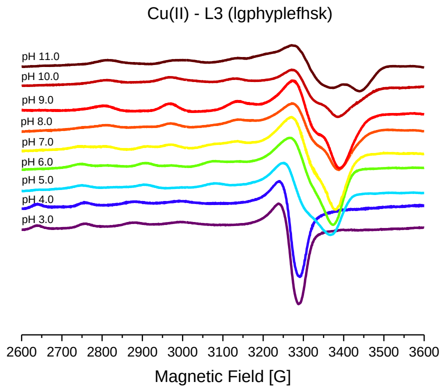

pH	$A_{\|\|}$ [G] (A_{zz})	$g_{\|\|}$ (g_{zz})	MW Frequency [GHz]	Suggested coordination mode
3.0	117.8	2.42	9.5193	4O
4.0	118.0	2.42	9.5192	4O
5.0	117.8<br>162.0	2.42<br>2.27	9.5211	4O + 2N
6.0	165.0<br>163.8	2.27<br>2.23	9.5188	2N + 3N
7.0	164.4<br>164.0	2.27<br>2.23	9.5195	2N + 3N
8.0	164.0<br>164.0	2.27<br>2.23	9.5220	2N + 3N
9.0	164.0	2.23	9.5200	3N
10.0	160.0	2.23	9.5160	3N
11.0	160.0<br>198.0	2.23<br>2.17	9.5217	3N + 4N

Figure S 7. pH-dependent EPR spectra for (A) Cu(II) – L1 (KSHFELPHYPGL); (B) Cu(II) – L2 (kshfelpyhpgl) and (C) Cu(II) – L3 (lgpyhplefhsk) systems in aqueous solution with the addition of ethylene glycol (30%) at temperature 77 K; [Cu(II)] = 0.001 M; molar ratio M:L equal to 0.8:1.

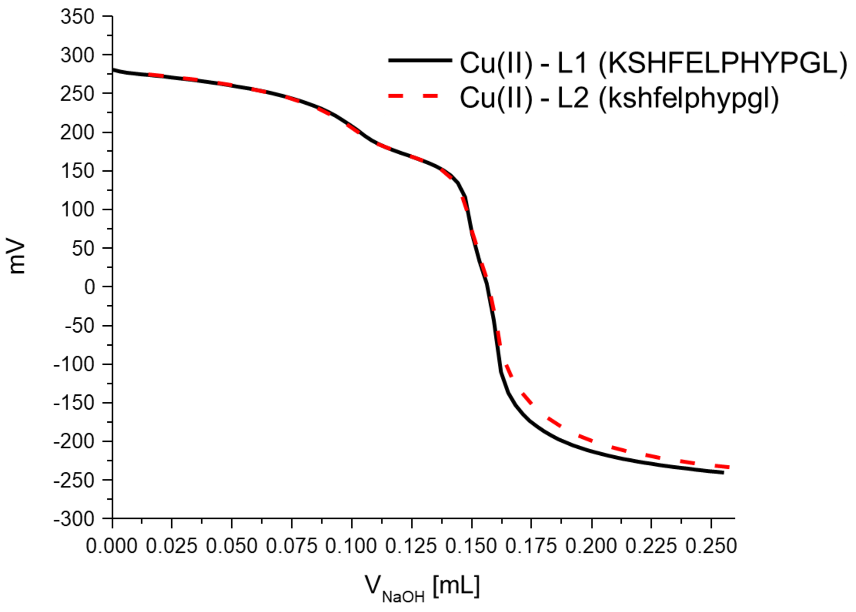

Figure S 8. The comparison of the potentiometric titration curves shape for the Cu(II) complexes with native peptide (L1, L-aa, black line) and D-amino acids analogue (L2, D-aa, red dotted line).

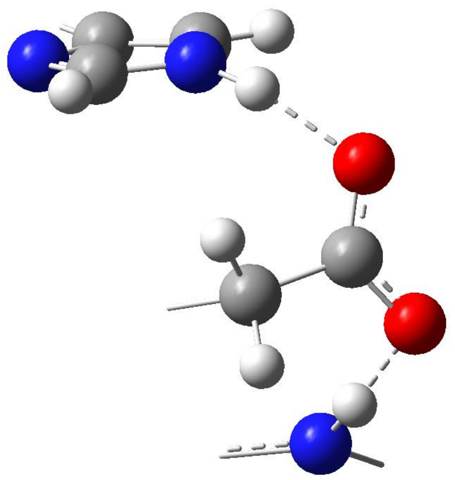

Figure S 9. The structure of hydrogen-bond interactions between the backbone of E5, the side chain of L6, and the side chain of H8 residues.

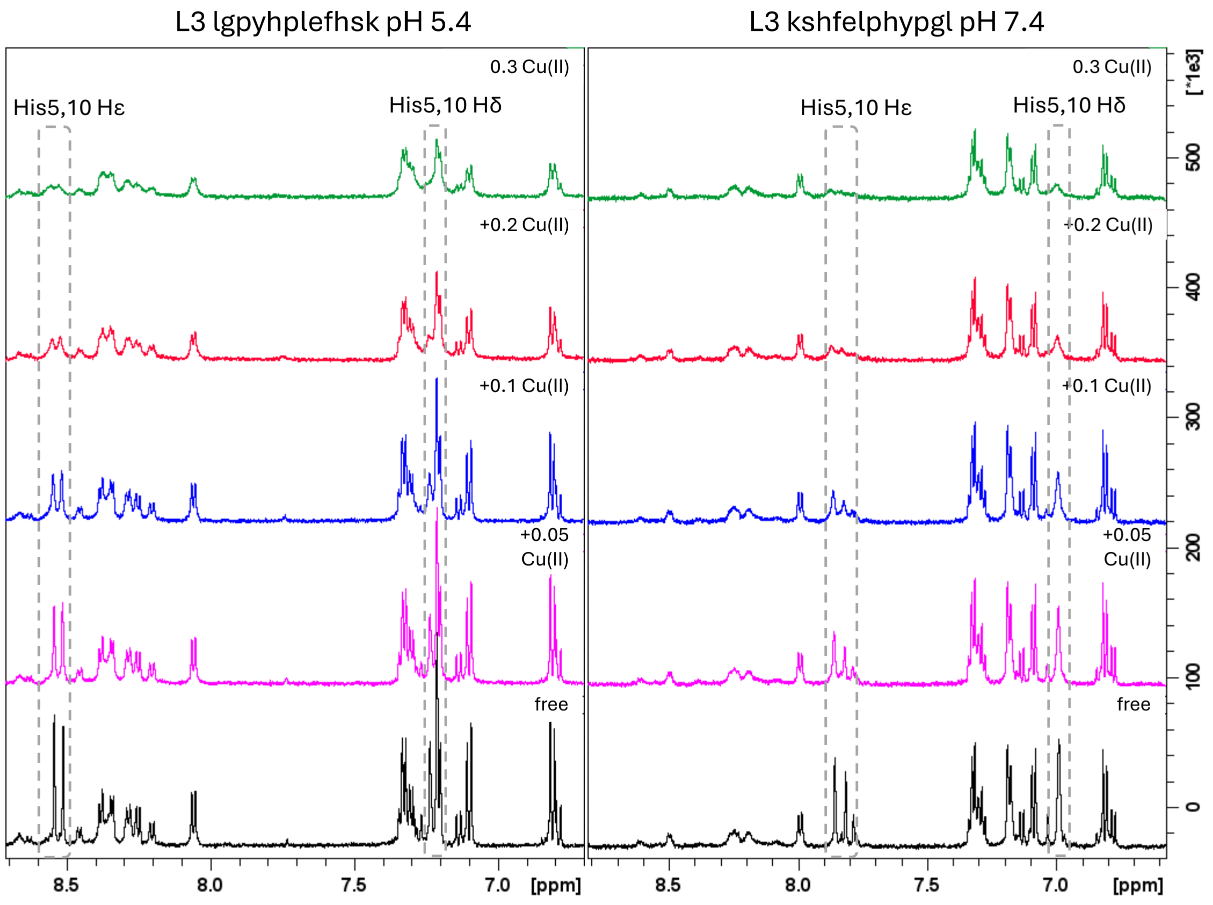

Figure S 10. ^{1}H NMR Cu(II) titrations of the L3 system at pH 5.4 (left) and 7.4 (right). The effects induced by increasing amounts of Cu(II) are shown in the aromatic region of the NMR spectra. [L3]= 0.5 mM. T=298 K. MES-d$_{13}$ 20 mM and phosphate buffer 20 mM. for pH 5.4 and 7.4. respectively.

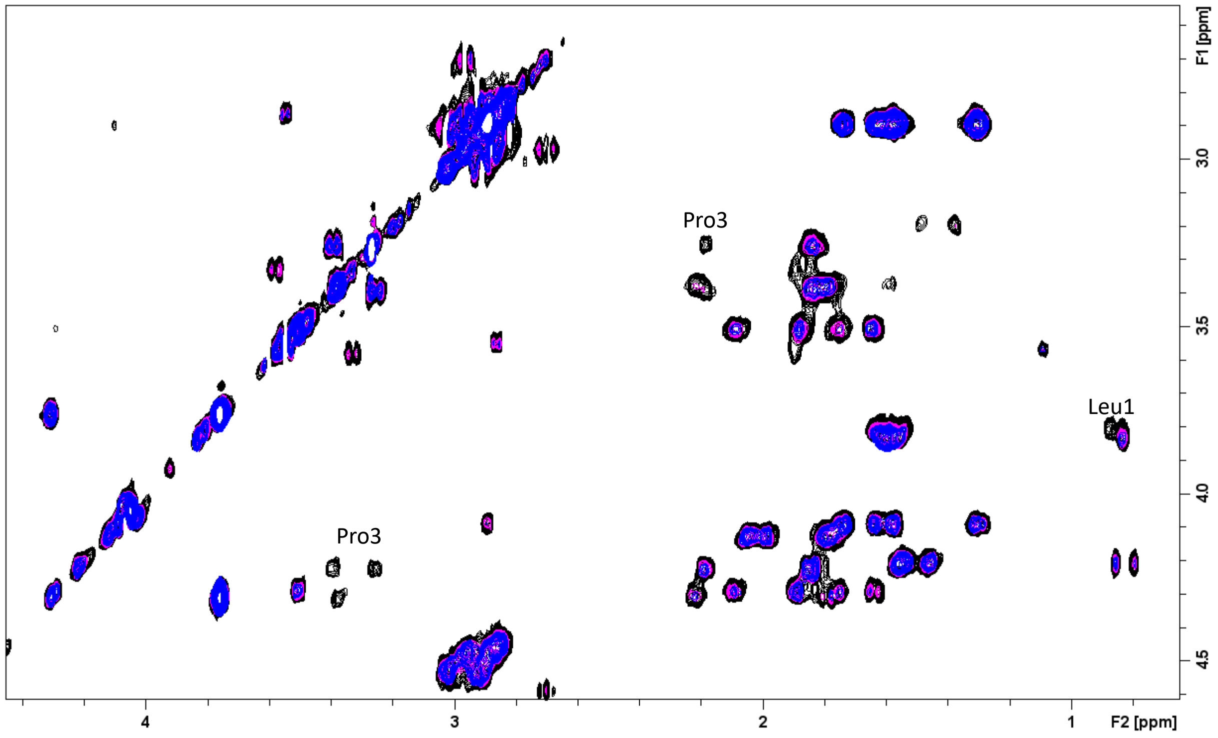

Figure S 11. Comparison of NMR ^{1}H-^{1}H TOCSY spectra of L3 (lgpyhplefhsk) 0.5 mM alone (black contours). in presence of 0.2 Cu(II) eqs. (magenta contours) and in presence of 0.3 Cu(II) eqs. (blue contours). T =298 K. pH 7.4. 20 mM phosphate buffer.

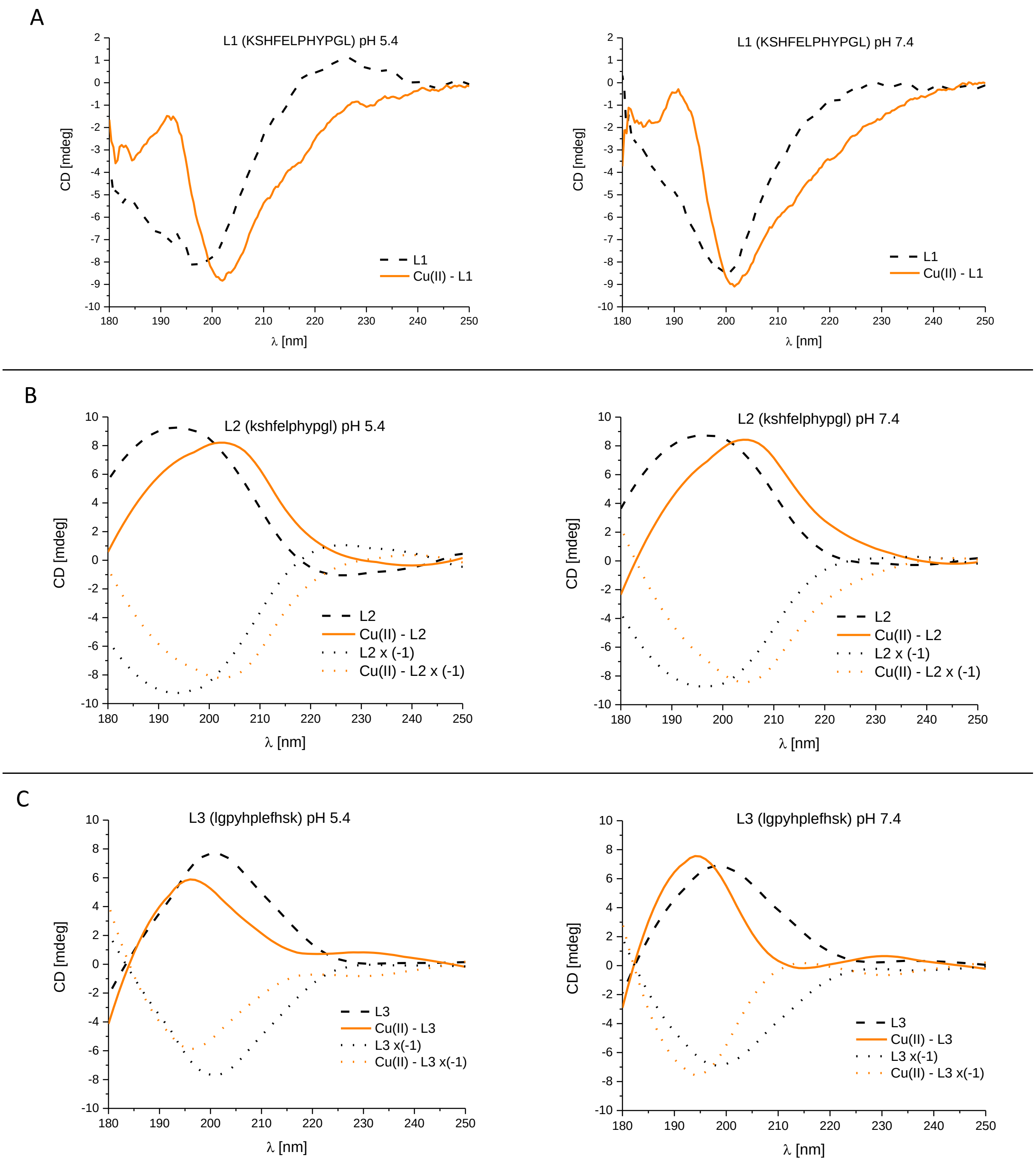

Figure S 12. CD spectra in the far-UV (180-250 nm) region at chosen pH values: 5.4 and 7.4 for the Cu(II) complexes with (A) L1 (KSHFELPHYPGL),(B) L2 (kshfelphypgl) and (C) L3 (lgpyhplefhsk) ligands in aqueous solution of 4 mM $HClO_4$ with I = 0.1 M $NaClO_4$; molar ratio M:L 0.8:1; the optical path length = 0.2 mm; C_L = 0.3 mM; dashed lines correspond to the ligand spectra; dotted lines correspond to recorded spectra with opposite sign for better comparison with the native - L-amino acid peptide.
